# Induced Dysbiosis Modifies Physiological Responses to Warming in a Temperate Sponge

**DOI:** 10.1111/1462-2920.70402

**Published:** 2026-09-06

**Authors:** Manon Broadribb, Torsten Thomas, Alice Rogers, James J. Bell

**Affiliations:** ^1^ School of Biological Sciences Victoria University of Wellington Wellington New Zealand; ^2^ Centre for Marine Science and Innovation School of Biological, Earth and Environmental Sciences, University of New South Wales Sydney Australia

**Keywords:** benthic ecosystems, climate change resilience, marine heatwaves, marine sponges, microbiome dysbiosis, physiological response, sponge‐microbe symbioses

## Abstract

Marine sponges host complex and specific microbiomes, yet the role of these microbial symbionts in sponge responses to acute environmental stress is not fully understood. We investigated how altering the microbiome influences physiological responses to temperature in the temperate calcareous sponge *Rowella lancifera* under simulated marine heatwave (MHW) conditions. Sponges were treated with broad‐spectrum antibiotics to disrupt their microbiome and then exposed to sub‐lethal MHW temperatures. Microbial community composition was analysed using 16S rRNA gene sequencing, including assessing diversity and differential abundance, while sponge physiological response to temperature was quantified via respiration rates. Antibiotic treatment significantly altered microbial community structure but did not independently induce a physiological response. Heat exposure increased respiration rates, with a stronger response observed in microbiome‐altered sponges. Multivariate analyses revealed a relationship between microbial community structure and respiration rate, suggesting an association between microbiome composition and host physiological responses. Overall, these findings indicate that sponge‐microbe symbioses may play a role in modulating physiological responses to increased temperatures. As MHWs become more frequent and severe under climate change, understanding sponge‐microbe partnerships will be important for predicting responses of sponges and the stability of benthic ecosystems.

## Graphical abstract

We investigated how microbiome disruption affects the physiological response of the calcareous sponge *Rowella lancifera* to simulated marine heatwave conditions. Antibiotic‐induced microbiome changes amplified physiological responses to increased temperatures, linking microbial stability to host performance. Results highlight that maintaining healthy sponge–microbe symbioses is important for future resilience under intensifying marine heatwaves.

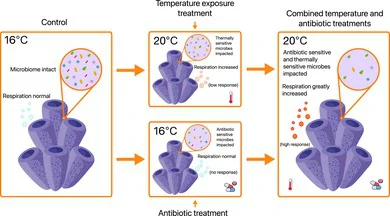

## 1 Introduction

Sponges are important members of benthic communities, with diverse functional roles in temperate, tropical, and polar ecosystems (Bell et al. 2023). As sessile filter feeders, they process large volumes of seawater, removing microorganisms, particulate organic matter (POM) and dissolved organic matter (DOM) (Pile and Young 2006; Lacoue‐Labarthe et al. 2016; Riisgård and Larsen 2022). This activity facilitates nutrient cycling, supports primary productivity, and mediates energy transfer between benthic and pelagic systems, known as the “sponge loop” (de Goeij et al. 2013; Rix et al. 2017). Many sponge species also function as ecosystem engineers by modifying substrates, stabilising sediments and providing complex habitats for other organisms (Bell et al. 2023; Schönberg 2016; Harris et al. 2021).

A defining feature of sponges is their close and often highly specific relationships with diverse microbial communities, including bacteria, archaea, and eukaryotic microbes (Thomas et al. 2016; Yang et al. 2019). The stability and specificity of these associations are central to sponge health and resilience, with microbial symbionts contributing significantly to metabolism and ecological success (Webster and Taylor 2012; Pita et al. 2018; Slaby et al. 2019). These symbionts perform essential metabolic functions such as nitrogen fixation, nitrification, denitrification, sulfur cycling, vitamin synthesis and secondary metabolite production (Webster and Taylor 2012; Fan et al. 2012; Lurgi et al. 2019; Díez‐Vives and Riesgo 2024).

Microbial symbionts can thus expand the metabolic capabilities of their hosts (González‐Pech et al. 2024), a phenomenon referred to as “phenotype expansion,” where host–microbe interactions enable organisms such as macroalgae and marine invertebrates to occupy a broader range of ecological niches (Parkinson and Baums 2014; DeWeese and Osborne 2021). Enhanced metabolic capacity may also improve tolerance to environmental stressors. For instance, shifts in microbial communities have been correlated with certain corals' ability to withstand extreme temperatures, ocean acidification, and pollution (Cameron et al. 2022; Pei et al. 2022; Wuitchik et al. 2024). However, manipulative experiments that go beyond correlative observation are currently lacking in marine invertebrates with the exception of corals (Ezzat et al. 2021; Connelly et al. 2022), and are needed to further establish and ascertain the role microbiomes play in the stress response of their hosts.

Marine heatwaves (MHWs) are increasing in frequency, duration and intensity due to anthropogenic climate change (Oliver et al. 2018). Their impacts span from individual species effects to ecosystem‐wide disruptions (Eakin et al. 2010; Thomsen et al. 2019). Sponges show a range of responses to increased temperatures, such as changes in respiration rate (Strano et al. 2022), spicule deformation (Ribeiro et al. 2021), reduced pumping (Massaro et al. 2012), bleaching and mortality (Bell et al. 2024) and importantly, shifts in microbial composition (Bell et al. 2024; Fan et al. 2013; Strano et al. 2023).

There is increasing evidence that environmental stressors can destabilise sponge–microbe symbioses, leading to microbial dysbiosis—a shift in community composition that can impair host health (Strano et al. 2023; Botté et al. 2023; de Castro‐Fernández et al. 2023). Dysbiosis may involve loss of microbial diversity, a decline in the abundance of core symbionts and/or the rise of opportunistic and pathogenic taxa (Posadas et al. 2022; Gastaldi et al. 2024; Efremova et al. 2024), undermining key functions and potentially increasing host susceptibility to disease (Slaby et al. 2019). Experimental disruption of the microbiome in sponges has been achieved using bacteriophages and antibiotics (Schmittmann et al. 2022; Steiner et al. 2024), though studies exploring how this influences physiological responses to temperature have not yet been performed. Understanding the role of microbial symbionts in the thermal tolerance of sponges is therefore critical to predicting their responses to future MHWs.

This study investigated the individual and combined effects of increased temperatures and experimentally induced microbiome disruption on respiration rate in the calcareous temperate sponge *Rowella lancifera*. This species was chosen due to its apparent resilience during the extreme 2022 MHWs in New Zealand, which caused bleaching and mortality in other sympatric sponges (Bell et al. 2024). We used broad‐spectrum antibiotics to first disrupt the microbiome and then compared the responses of microbiome‐intact and microbiome‐disrupted individuals under simulated MHW conditions. We hypothesised that: (1) Antibiotics would significantly alter the microbiome; (2) sponges with intact microbiomes would exhibit measurable physiological responses under simulated MHW conditions; and (3) these responses would be exacerbated in microbiome‐disrupted individuals. We assessed microbial community changes following antibiotic and heat treatment and measured sponge respiration rates as changes in respiration are widely used in the literature to quantify physiological responses to stressors, including thermal exposure, in sponges (Strano et al. 2022; Bennett et al. 2017; Ramsby et al. 2018; Bosch‐Belmar et al. 2023; Beepat et al. 2021). Because metabolic rates are intrinsically temperature‐dependent in ectotherms (Brown et al. 2004), increased respiration reflects elevated metabolic demand and may indicate stress when temperatures approach physiological limits. Combined, we aimed to evaluate the importance of dysbiosis in sponge responses to elevated temperature.

## 2 Methods

### 2.1 Sponge Collection and Maintenance

*R. lancifera* sponges (*n* = 36) of roughly the same size (~5 cm length, ~2 cm diameter) were collected using SCUBA from approximately 15 m depth at Bradshaw Sound, Doubtful Sound, Southern New Zealand (45°17.103′ S 167°02.034′ E; Figure S1) on the 13th of January 2024. Dive knives were used to pry the sponges gently from the substrate whilst keeping the base intact to minimise damage to the sponge. Sponges were then placed into zip lock bags whilst still underwater to ensure no contact with the air, and transferred into thermally insulated bins after the dive was completed. The bins contained seawater collected from the sampling site and were set up with aquarium aerators to ensure the water remained oxygenated, with temperature monitored and regulated at 16°C–17°C, and water changed regularly throughout transportation. On the following day sponges were transferred into large carrying containers, again with fresh seawater, and secured in a cooler for transportation back to the Victoria University Coastal Ecology Laboratory (VUCEL), a roughly 6 h journey.

Once they arrived at VUCEL, sponges were placed into 4 L tanks (*n* = 3 per tank) and attached to small ceramic tiles using aluminium wire, ensuring they were attached in an upright position with the base of the sponge on the tile. As sponges feed on POM, DOM and pico‐, nano‐ and microplankton (Ribes et al. 1999), tanks were set up to allow a flow of 10 μm filtered seawater for the duration of the acclimation and experimental periods (flow rate: 12 ± 1 L h^−1^). Water temperature in the tanks during the acclimation period was set to 16°C for all tanks to match the water temperature that the sponges were collected from. Sponges were allowed to acclimate to laboratory conditions for 14 days before the experiment began. Tanks were opaque with lids to minimise any impacts of light and external contamination.

### 2.2 Experimental Conditions

**FIGURE 1:**
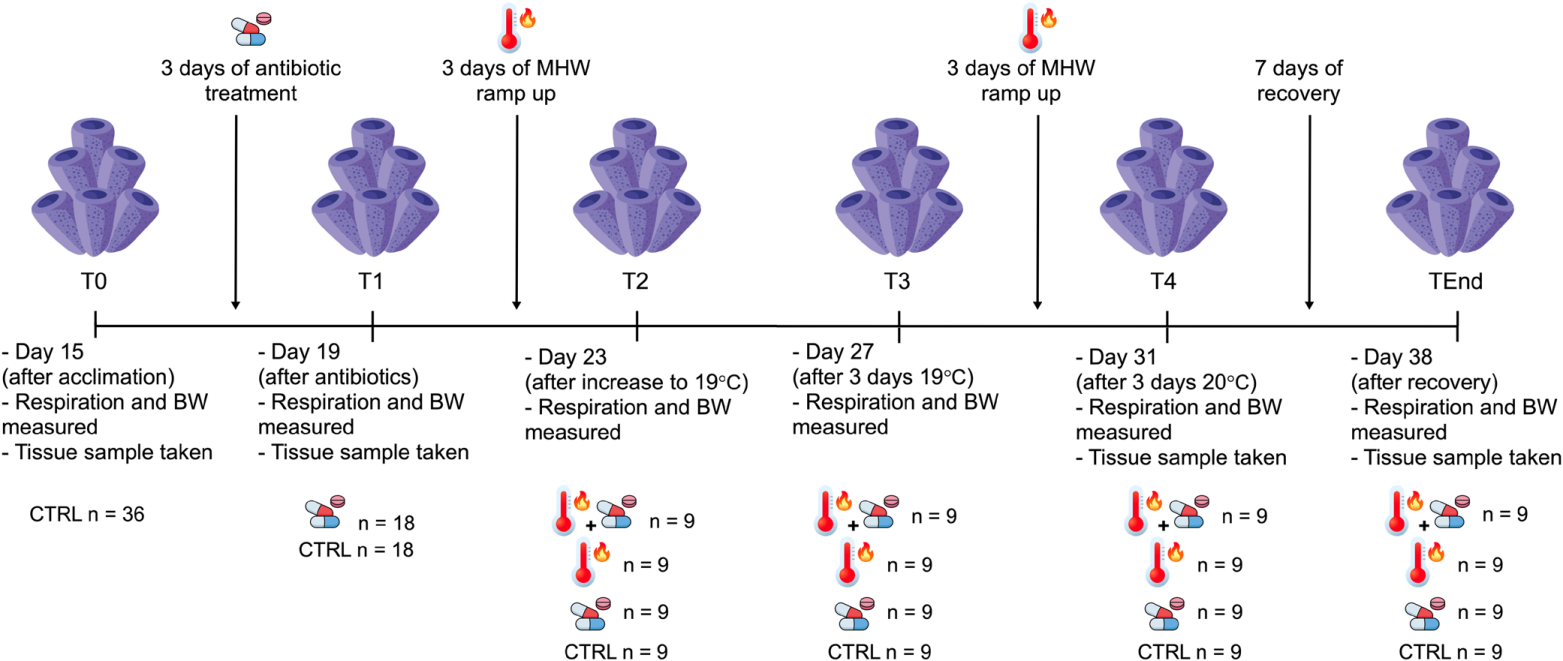
Schematic representation of the experimental timeline, showing days during which different treatments were administered, and days during which physiological measurements and tissue samples were taken.

Three experimental treatments (heatwave, antibiotics, and antibiotics + heatwave) and a control were designed to examine the separate and interactive effects of simulated MHW conditions and artificial microbiome disruption on the physiological responses of *R. lancifera* (Figure 1). *N* = 3 sponges were placed in 12 4 L treatment tanks each containing a small aquarium pump (1.5 W, 180 L/h flow, AYNEFY, Shenzhen, China) to keep water circulating throughout the experiment. Tanks were kept in 40 L water baths (*n* = 3 tanks per water bath) that contained two large pumps to circulate water (10 W, 700 L/h flow, Jebao, China). Water temperature in the water baths was controlled by an aquarium controller system (Apex Classic, Neptune Systems, Morgan Hill, CA, USA), connected to aquarium heaters (200 W, Eheim, Germany) and two chillers (HC‐500A, Hailea, China) (Figure S2). Water temperature of the individual treatment tanks was also checked twice daily and the water baths once daily by a YSI Pro30 probe (Total Lab Systems, Auckland, NZ) and the control system was calibrated accordingly.

### 2.3 Antibiotic Treatment

After the initial acclimation phase of 14 days, antibiotics were administered (Figure 1). For the antibiotic treatment, 6 tanks from the same two water baths (to avoid any contamination) were treated with antibiotics at the following concentrations: nalidixic acid (200 mg/L), neomycin (200 mg/L), ampicillin (200 mg/L), rifampicin (50 mg/L) and polymyxin B (8 mg/L). These antibiotics and concentrations were based on a previous study aiming to disrupt the microbiome of the sponge *Halichondria panicea* (Schmittmann et al. 2022), and their efficacy was tested on *R. lancifera* in preliminary laboratory trials. Antibiotics were administered in solution once per day for three days at 7 am, before which the inflow to all tanks including controls was turned off, and the outflow blocked to keep the antibiotic solution within the tank. The inflow was turned back on at 7 pm, and outflow released, which gave 12 h of antibiotic treatment followed by 12 h of water flow before the process was repeated. To ensure that 12 h without flow would not cause sponges to be re‐filtering waste products, we carried out preliminary trials to calculate average pumping rate for *R. lancifera* and used this to infer the minimum time it would take one sponge to filter 4 L of water (Table S1).

### 2.4 MHW Treatment

Following the antibiotic treatment, *n* = 6 untreated sponges and *n* = 6 antibiotic‐treated sponges were exposed to a simulated MHW peaking at 20°C (Figure 1). This peak was based on consecutive recent heatwaves in the Fiordland Marine Area in the summers of 2022 and 2023 which peaked at 20.3°C and 19.95°C, respectively (Figure S2), and a previous study which found 20°C to be a sublethal, response‐inducing temperature for *R. lancifera* (Broadribb et al. 2026). Temperature was first raised by 1°C per day for 3 days to 19°C. Treatment tanks were then kept at 19°C for 3 days, and finally the temperature was increased by a further 1°C (over one day) to reach the peak of the simulated MHW at 20°C. Temperatures in treatment tanks remained at 20°C for 3 days. The temperature was then dropped by 2°C per day for 2 days to 16°C where it remained for the duration of the recovery period (6 days). Daily temperature regimes are listed in Table S2. Buoyant weight and respiration rates of all sponges were measured on T0, T1, T2, T3, T4 and TEnd (Figure 1).

### 2.5 Microbial Community Analysis

Samples of all sponges (0.5 cm^3^) were taken with a sterile scalpel and forceps at T0, T1, T4, TEnd (Figure 1) before being placed in DESS (DMSO‐EDTA‐saturated salt solution) and stored at −80°C. DNA was then extracted using the DNEasy PowerSoil Pro Kit following the manufacturer's instructions (Qiagen, Hilden, Germany). Nanodrop and gel electrophoresis on 1% agarose were used to assess the concentration and check the quality of the extracted DNA.

Amplicon library construction and sequencing were performed by the Ramaciotti Centre for Genomics at the University of New South Wales, Australia on the MiSeq V2 platform (Illumina). To target the V4 region of the 16S rRNA gene of bacteria and archaea, the modified primers 515f (5′‐GTGYCAGCMGCCGCGGTAA‐3′) (Parada et al. 2016), and 806r (5′‐GGACTACNVGGGTWTCTAAT‐3′) (Apprill et al. 2015), containing Illumina overhang adapter sequences were used. For library preparation, extracted DNA was PCR amplified with 12.5 μL of EconoTaq PLUS GREEN 2X Master Mix (Lucigen, Middleton, WI, USA) 1 μL of each primer (10 μM) and 2 μL of DNA in a 25 μL final reaction solution. PCR conditions were 94°C for 3 min for initial denaturation, followed by 30 cycles of denaturation at 94°C for 45 s, annealing at 50°C for 60 s, and elongation at 72°C for 90 s, and a final extension at 72°C for 10 min. PCR products were run on a DNA gel to confirm correct amplification and cleaned with AMPure XP beads (Beckman Coulter, Brea, CA, USA). An indexing PCR was carried out with Illumina DNA/RNA UD indexes with the following conditions: Activation at 95°C for 3 min, followed by 8 cycles of 95°C for 30 s, 55°C for 30 s, and 72°C for 30 s, and a final extension at 72°C for 5 min. After the indexing PCR, a gel was run for quality control and all samples were normalised using a SequelPrep normalisation kit (Thermo Fisher, Waltham, MA, USE). An equal volume was taken from all samples and pooled, the pool was cleaned with AMPure XP beads and quantified with Qubit and a Tapestation before finally being sequenced with a Miseq v2 2x250bp run.

Raw sequences were filtered, trimmed, and primers were removed using USEARCH v.11.0.667 and Trimmomatic v.0.38 as previously described by Strano et al. (Strano et al. 2023). Amplicon sequence variants (ASVs) were generated using UNOISE3, and chimeras were removed with UCHIME2 in reference mode using the SILVA database release 132. For taxonomic assignment of ASVs, the Bayesian Last Common Ancestor (BLCA) algorithm was applied with SILVA release 132 (Gao et al. 2017; Quast et al. 2013; Yilmaz et al. 2014).

### 2.6 Buoyant Weight and Respiration Rate

Buoyant weight was measured in order to standardise sponge respiration rate using a digital scale (A&D FX‐200i) following the methods of Osinga (Osinga et al. 1999) whereby the scale was placed on a platform above a basin filled with seawater at the corresponding treatment temperature, and a hanger attached to the scale in such a way that the sponge placed on the hanger below the scale would remain fully submerged during weighing. At the end of the experiment the surviving sponges (*n* = 29) were dried for 72 h at 60°C and then combusted at 500°C for five hours. A linear regression was then carried out to convert previous buoyant weight measurements to ash‐free dry weight (AFDW) (Figure S4).

Respiration rates were measured as per Strano et al. (Strano et al. 2023). Nine sponges per measurement round (all sponges from the same treatment or control to avoid potential contamination) were separately placed in sealed 250 mL cylindrical glass respiration chambers with oxygen sensor spots (PreSens SP‐PSt3‐NAU, Regensburg, Germany) attached to their inner surface. A submersible magnetic stirrer maintained continual water movement inside the respiration chambers, and chambers were maintained in a thermally controlled water bath with temperatures corresponding to given experimental conditions for the duration of the respiration measurements. After 15 min of acclimation (which was a sufficient period to acclimatise sponges according to preliminary trials), oxygen concentration inside the chambers was measured every 15 min for 45 min, using a Fibox 4 oxygen probe (PreSens). To record possible microbial respiration in seawater, one blank measurement of seawater alone was included for every incubation. Sponge respiration rate was then standardised to sponge ash‐free dry weight (mgO_2_ gAFDW^−1^ h^−1^), as suggested by Bell et al. (Bell et al. 2020).

### 2.7 Survival and Health Monitoring

Sponges were monitored daily for the duration of the experiment. Sponges showing signs of necrosis and/or severe tissue degradation were removed from their tanks to ensure they did not impact other sponges in the experiment. This resulted in the removal of six sponges throughout the experimental period (Control = 1, Antibiotic = 1, Heatwave = 3, Antibiotic + Heatwave = 1).

### 2.8 Statistical Analyses

All statistical data analyses were performed in R version 4.3.3 (R Core Team 2024). For multiple comparisons, *p*‐values were corrected using the Benjamini‐Hochberg (BH) procedure (Benjamini and Hochberg 1995). The corrected *p*‐values were used to determine statistical significance at an alpha level of 0.05. Plots were created using ‘ggplot2’ (Wickham 2016).

At T1, after antibiotic treatment but before heatwave treatment, we grouped together sponges in control and heatwave tanks as a control group, and antibiotic and antibiotic + heatwave tanks as solely antibiotic‐treated sponges. ASV data was normalised using total sum scaling in order to give relative microbial abundances. Differences in microbial communities were visualised using nMDS (non‐multidimensional scaling) plots (R package *vegan*) (Oksanen et al. 2025), based on Bray‐Curtis dissimilarities (adonis2, *vegan*) (Oksanen et al. 2025). Statistical differences in community structure were then analysed using permutational multivariate analysis of variance (PERMANOVA) (Anderson 2001), and differences in dispersion of communities around their group centroids were evaluated using permutational multivariate analysis of dispersion (PERMDISP) (Anderson 2006), in both cases using 9999 unrestricted permutations. Alpha diversity indices of microbial communities (Peilou's evenness, ASV richness, and Shannon diversity) were calculated on read numbers rarefied to the minimum sequencing depth using 100 iterations, via the *phyloseq_mult_raref_div* function (R package *metagMisc*) (Mikryukov 2020). Differences in diversity indices between treatments at each time point were then explored using linear models and estimated marginal means, on data which was square root transformed to fit normality assumptions (*lm*, r package *stats* (R Core Team 2024); *emmeans*, r package *emmeans* (Lenth 2025)). To identify significant changes in the abundance of specific microbial families the ANCOM‐BC2 package was used on raw ASV data (Lin and Peddada 2023).

For respiration rate analysis we used a linear mixed‐effects model with normally distributed errors and random intercepts (lmer, *lme4* package) (Bates et al. 2015). In the mixed models, treatment and time were considered fixed effects, whilst experimental tank and sponge individual were considered random effects. The experimental tank effect was included to address pseudo‐replication. For these models, fixed‐ and random‐effect terms were tested using the function anova and ranova (R package *lmerTest*) (Kuznetsova et al. 2017), respectively, whilst *post hoc* pairwise comparisons were computed on estimated marginal means using emmeans (R package *emmeans*) (Lenth 2025). Respiration rates were log (x + 1) transformed to meet normality assumptions. The goodness of fit, normality and homoscedasticity of the errors were checked for the model by inspecting plots of the normalised residuals and the quantile‐quantile plots.

We used the *mvabund* package in R (Wang et al. 2012) to assess the relationship between ASV counts and respiration rate across all sponge individuals. Low‐abundance ASVs were filtered based on prevalence (< 10% of samples) and total read counts (< 200). A negative binomial manyglm was fitted using log(x + 1) transformed respiration rate as the predictor to reduce skew and improve model fit. Significance was tested via likelihood‐based methods, and ASV‐level *p*‐values were BH corrected. To validate these findings across taxonomic resolution, the analysis was repeated at the family level by aggregating ASV counts into family‐level abundances and applying the same *mvabund* workflow.

## 3 Results

### 3.1 Impact of Antibiotics and MHW on Microbial Communities

In order to define the impact of antibiotic and temperature treatments on the bacterial and archaeal communities of *Rowella lancifera* (Figure 1), we performed 16S rRNA gene sequencing. A total of 44,431,304 raw reads, 2,443,917 clean reads (with 181,554 ± 67,636 as mean and s.d. of reads per sample) and 1466 ASVs were obtained for the V4 region of the 16S rRNA gene from sponges under each treatment at T0, T1, T4 and TEnd (Figure S5).

PERMANOVA showed microbial community structure to be affected by both treatment and time, with a significant interaction effect (Table 1). We found microbial communities differed significantly between at least two time points under all treatments, including under control conditions (Table S5; Figure S7). For this reason, moving forward we only discuss results from analyses between treatments at each time point, and not within treatments through time.

**TABLE 1:** PERMANOVA table of results showing effect of time, treatment, and their interaction on microbial community structure of sponges. Model was run on Bray‐Curtis dissimilarities using 9999 permutations on log transformed data.

	DF	SS	MS	Pseudo‐F	R2	*p*
Treatment	3	1.8187	0.6062	6.8050	0.2042	0.0001**
Time	2	1.0204	0.5102	5.7270	0.1146	0.0001**
Treatment × Time	4	0.6320	0.1580	1.7735	0.0710	0.0121*
Residuals	61	5.4343	0.0891		0.6102	
Total	70	8.9054			1.0000	

* *p* < 0.05.

** *p* < 0.01.

**FIGURE 2:**
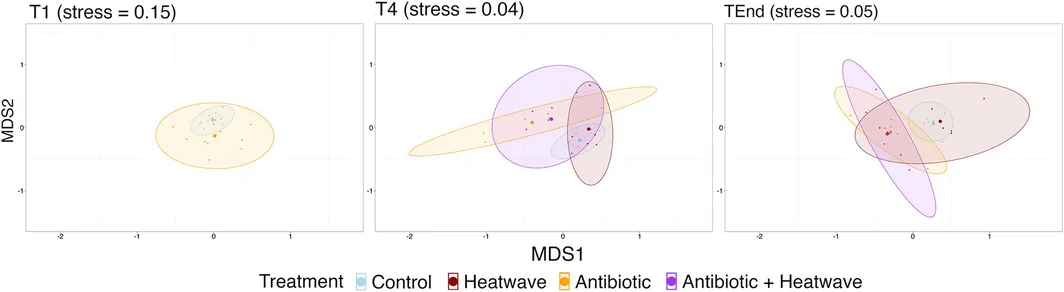
Non‐metric multidimensional scaling (nMDS) plot of microbial communities under different treatments at each time point. Large points represent group centroids, small points represent individual samples, and ellipses represent 95% confidence around the centroid. NB at T1 after antibiotic treatment, but before temperature treatment, control and heatwave sponges were grouped as ‘control’, and antibiotic and antibiotic + heatwave sponges were grouped as ‘antibiotic’.

When looking at individual time points, microbial community structure was affected by treatment at T1, T4 and TEnd (Table S3, Figure 2). At T0, after acclimation and before any treatment had been administered, microbial communities did not differ between sponges in any treatment tanks (Table S3). At T1, after antibiotic treatment, microbial communities differed significantly between control and antibiotic‐treated sponges (Table S3). PERMDISP results showed significant differences in dispersion of microbial communities around their group centroids between control and antibiotic‐treated sponges (Table S4; Figure 2 and Figure S6), meaning significant PERMANOVA results at this time point were potentially due to differences in dispersion.

Post hoc pairwise analysis showed that at T4, microbial community structure differed significantly between all treatments except between antibiotic and antibiotic + heatwave, and between control and heatwave‐treated sponges (Figure 2; Table 2). At this time point, divergence in community composition was greater between control and antibiotic‐treated sponges compared to T1 (Figure 2; Table 2 and Table S3). At TEnd, significant differences were observed between control and antibiotic, control and antibiotic + heatwave, antibiotic and heatwave, antibiotic + heatwave and heatwave, and control and heatwave‐treated sponges. No significant difference was found between antibiotic and antibiotic + heatwave treatments (Figure 2; Table 2). No differences in dispersion of microbial communities from their group centroids between treatments were detected at T1 or TEnd (Table S4; Figure S6).

**TABLE 2:** Post hoc pairwise analyses of PERMANOVA results showing differences in microbial community composition between treatments at T4 and TEnd.

Time	Pairs	DF	SS	F value	R^2^	*p* (BH)
T4	Antibiotic vs. Antibiotic + Heatwave	1	0.1112	0.8431	0.0778	0.5092
	Antibiotic vs. Control	1	0.4472	4.5473	0.3126	0.0226*
	Antibiotic vs. Heatwave	1	0.4935	4.1911	0.2953	0.0226*
	Antibiotic + Heatwave vs. Control	1	0.3170	4.0254	0.2870	0.0234*
	Antibiotic + Heatwave vs. Heatwave	1	0.2983	3.0389	0.2331	0.0226*
	Control vs. Heatwave	1	0.1269	1.9636	0.1641	0.0715
TEnd	Antibiotic vs. Antibiotic + Heatwave	1	0.0822	0.6586	0.0618	0.7363
	Antibiotic vs. Control	1	0.4460	5.6739	0.3620	0.0060**
	Antibiotic vs. Heatwave	1	0.5142	4.4668	0.3088	0.0206*
	Antibiotic + Heatwave vs. Control	1	0.5198	5.6331	0.3603	0.0060**
	Antibiotic + Heatwave vs. Heatwave	1	0.4683	3.6362	0.2667	0.0206*
	Control vs. Heatwave	1	0.1562	1.8922	0.1591	0.0409*

* *p* < 0.05.

** *p* < 0.01.

**FIGURE 3:**
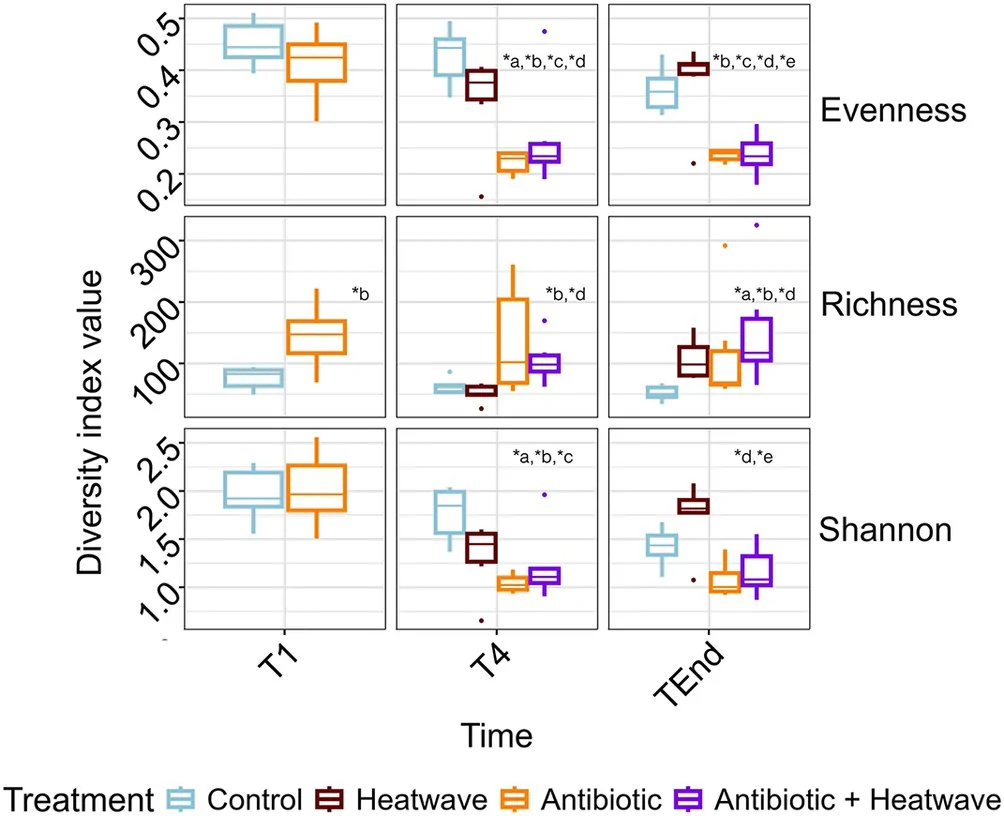
Box and whisker plot of median (with 25th, 50th and 75th percentile, plus outliers), species richness, Pielou's evenness, Shannon diversity and Simpson's diversity (based on samples rarefied to the minimum read depth) of sponge microbial communities at different time points throughout the experiment, split between treatments. *a = control and heatwave treatments differ significantly, *b = control and antibiotic treatments differ significantly, *c = control and antibiotic + heatwave treatments differ significantly, *d = heatwave and antibiotic treatments differ significantly, and *e = heatwave and antibiotic + heatwave treatments differ significantly.

Linear mixed effect models found that treatment, and the interaction between treatment and time, significantly affected ASV richness, whereas time alone had no effect (Table 3). Pielou's evenness and Shannon diversity were affected by time, treatment, and their interaction (Table 3). ASV richness was significantly lower in antibiotic‐treated sponges compared to controls at T1, T4, and TEnd, and lower than heatwave‐treated sponges at T4. At TEnd, ASV richness was also significantly lower in heatwave and antibiotic + heatwave treated sponges compared to controls (Figure 3; Table S6). Pielou's evenness was significantly higher in antibiotic, heatwave, and antibiotic + heatwave treated sponges when compared to controls at T4, and remained higher in antibiotic‐treated sponges compared to heatwave‐treated sponges at T4 and TEnd. At TEnd, evenness was also higher in antibiotic + heatwave treatments compared to control and heatwave treatments (Figure 3; Table S7). Shannon diversity did not differ between control and antibiotic treatments at T1, but was higher in heatwave, antibiotic, and antibiotic + heatwave treatments at T4. At TEnd, Shannon diversity was higher in antibiotic and antibiotic + heatwave treatments compared to heatwave alone (Figure 3; Table S8).

**TABLE 3:** Results from linear mixed effect models showing effects of time, treatment and their interaction on (A) species richness, (B) Pielou's evenness, and (C) Shannon diversity of sponge microbial communities.

	DF	SS	MS	F value	*p*
(A) Richness					
Treatment	3	53.5884	17.8628	6.1631	0.0013**
Time	2	17.9874	8.9937	3.1030	0.0546
Treatment × Time	4	35.8656	8.9664	3.0936	0.0256*
(B) Evenness	DF	SS	MS	F value	*p*
Treatment	3	0.1852	0.0617	22.9104	0.0001**
Time	2	0.1285	0.0643	23.8497	0.0001**
Treatment × Time	4	0.0535	0.0134	4.9626	0.0016**
(C) Shannon	DF	SS	MS	F value	*p*
Treatment	3	0.2995	0.0998	8.1149	0.0003**
Time	2	0.8109	0.4054	32.9583	0.0001**
Treatment × Time	4	0.3360	0.0840	6.8287	0.0002**

* *p* < 0.05.

** *p* < 0.01.

### 3.2 Taxonomic Changes in Microbiome During MHW Differ With Antibiotic Treatment

**FIGURE 4:**
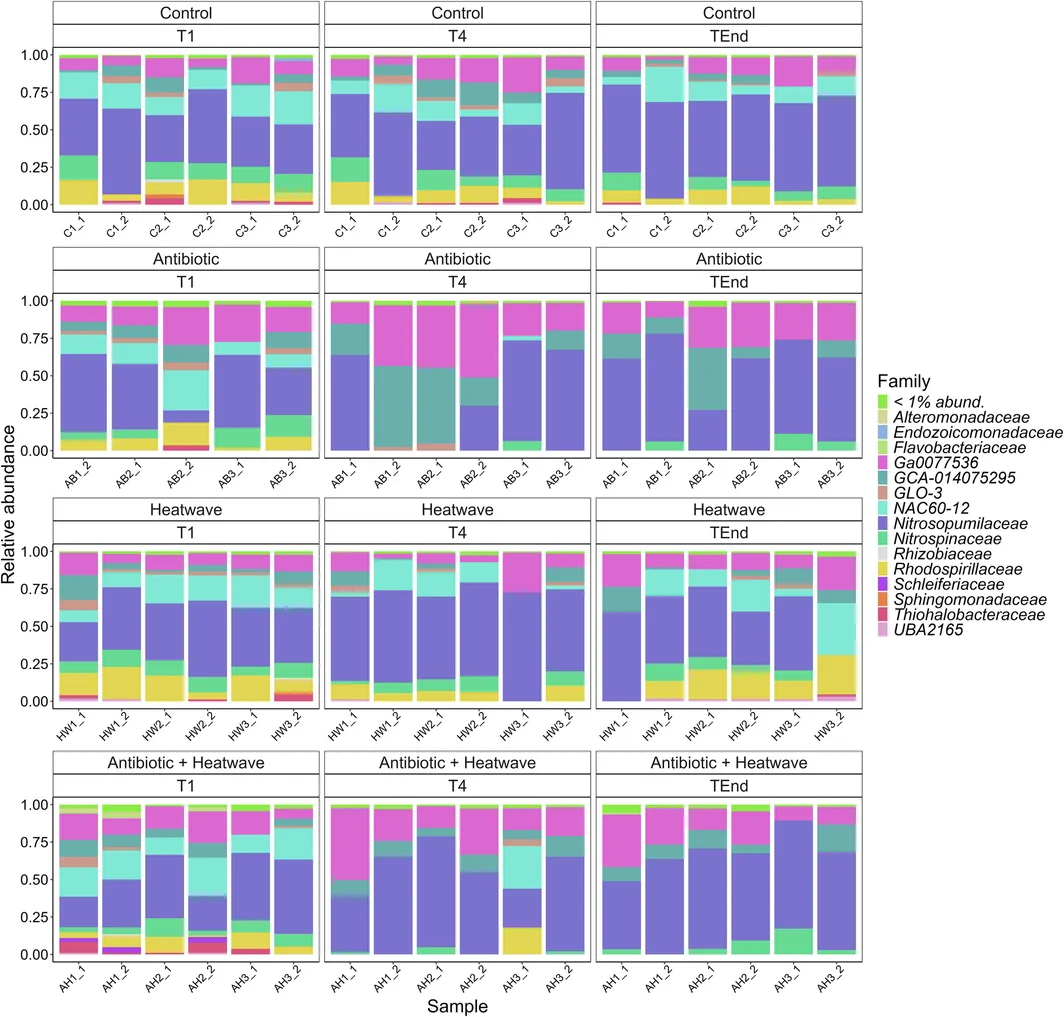
Relative read abundance of the most abundant microbial families at each time point under each treatment. It should be noted that at T1, antibiotic treatment had been administered, but there had been no heatwave treatment, so “heatwave” sponges are under the same conditions as control sponges at this time point, and “antibiotic + heatwave” sponges were under the same conditions as antibiotic‐treated sponges at this time point.

At all timepoints, under all treatments, the microbial family with the highest relative read abundance was *Nitrosopumilaceae*, making up between 32.6% and 62.0% of the total reads. At T1, the microbial family with the second highest relative read abundance under control conditions was *Rhodospirillaceae*, making up between 10.7% and 13.9% of total reads, whereas under antibiotic treatment this was *Ga0077536*, making up between 14.5% and 18.0% of the total reads. Other abundant families across the majority of times and treatments included *Nitrospinaceae, GCA‐014175295, GLO‐3* and *Thiohalobacteraceae* (Figure 4).

**FIGURE 5:**
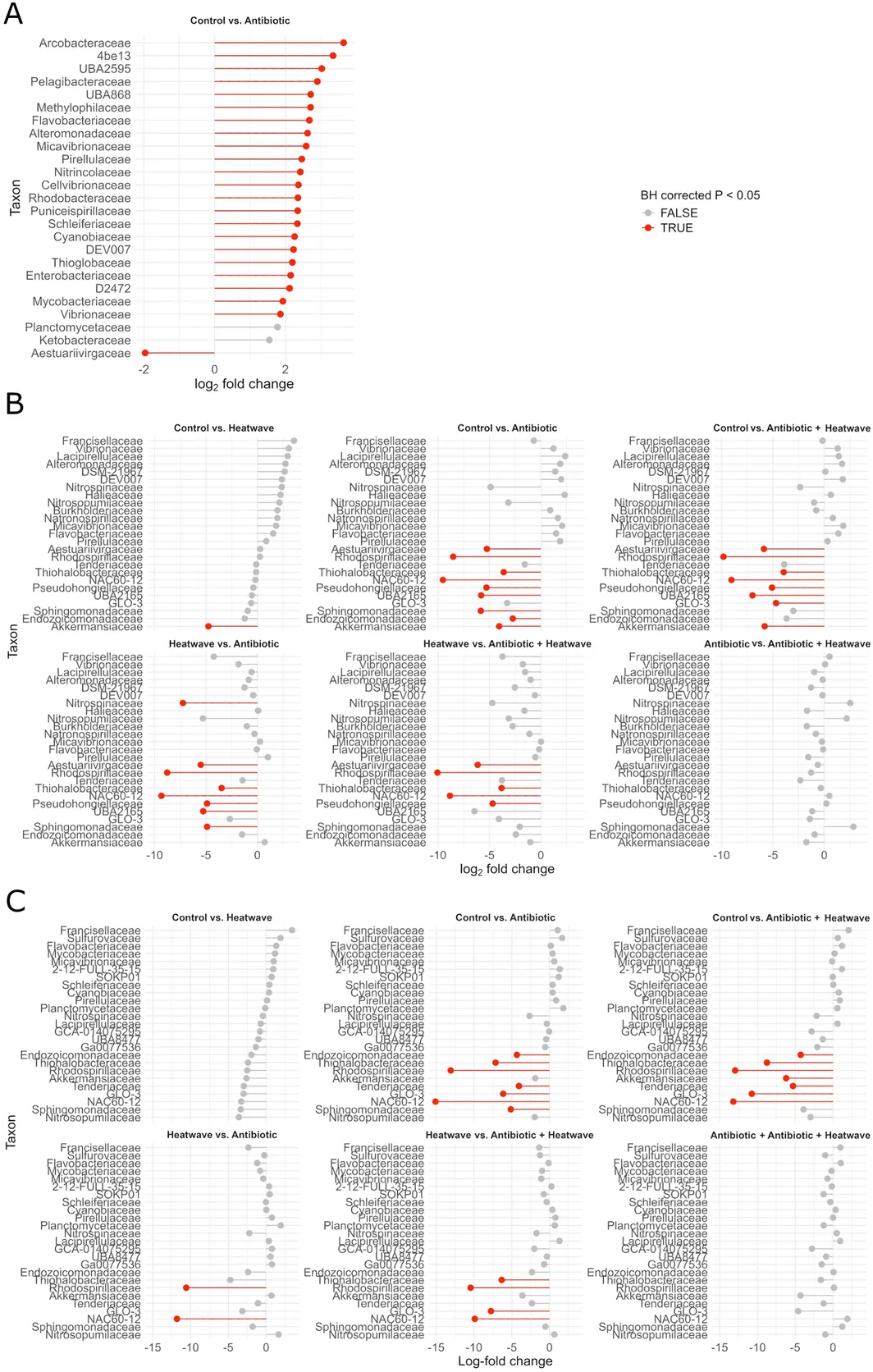
Results from ANCOMBC analysis showing differentially abundant microbial families between treatments at (A) T1, (B) T4 and (C) TEnd. Log fold changes are on the natural log scale and indicate changes in relative read abundance. NB at T1 after antibiotic treatment, but before temperature treatment, control and heatwave sponges were grouped as ‘control’, and antibiotic and antibiotic + heatwave sponges were grouped as ‘antibiotic’, so A shows differences between only control and antibiotic‐treated sponges.

In terms of changes in relative abundances, after antibiotic treatment but before heatwave treatment at T1, ANCOM‐BC2 analysis showed significant differences in the relative read abundance of 23 microbial families between control and antibiotic‐treated sponges. Of these, the ones with the highest relative read abundances were *Flavobacteriaceae, Schleiferiaceae, Alteromonadaceae, Rhodobacteraceae, UBA868, Pelagibacteraceae, Pirellulaceae* and *Enterobacteriaceae* (Figure 4). All families which differed significantly at this time point increased in relative read abundance in the antibiotic‐treated samples, except *Aestuariivirgaceae*, which decreased (Table S9; Figure 5).

At T4, there were nine microbial families which differed in relative read abundance between control and antibiotic‐treated sponges, however none of these except *Aestuariivirgaceae* were the same families which had differed at T1, and conversely to T1 no families significantly increased in relative read abundance under antibiotic treatment, but instead all decreased (Table S10; Figure 5). Of those which differed, the families with the highest relative read abundance were *Rhodospirillaceae, NAC60‐12* and *Thiohalobacteraceae* (Figure 4). Only one microbial family, *Akkermansiaceae*, differed significantly in relative read abundance between control and heatwave conditions at T4, and decreased under heatwave conditions (Table S10; Figure 5). The microbial families which differed under antibiotic + heatwave treatment when compared to control conditions were the same as those which differed under antibiotic treatment alone, except *Sphingomonadaceae* and *Endozoicomonadaceae*, which did not differ under heatwave + antibiotic treatment yet decreased under antibiotic treatment alone, and *GLO‐3*, which did not differ under antibiotic treatment alone but decreased in relative read abundance under antibiotic + heatwave conditions (Table S10; Figure 5).

At TEnd (i.e., after recovery), there were fewer microbial families that differed in relative read abundance between treatments when compared to previous time points, suggesting recovery from dysbiosis and heat treatment. Interestingly, *Akkermansiaceae* no longer differed between control and heatwave or control and antibiotic‐treated sponges, however it was still significantly less abundant in antibiotic + heatwave treated sponges when compared to control sponges, suggesting slower recovery under combined treatments (Table S12; Figure 4). Additionally, the family *Tenderiaceae* had a significantly lower read abundance at this time point under antibiotic and antibiotic + heatwave conditions when compared to control sponges, despite not having differed significantly between any treatments at previous time points, pointing to some latent effects of the antibiotic treatment (Table S12; Figure 4).

### 3.3 Respiration Analysis Shows Differential Response During MHW

**FIGURE 6:**
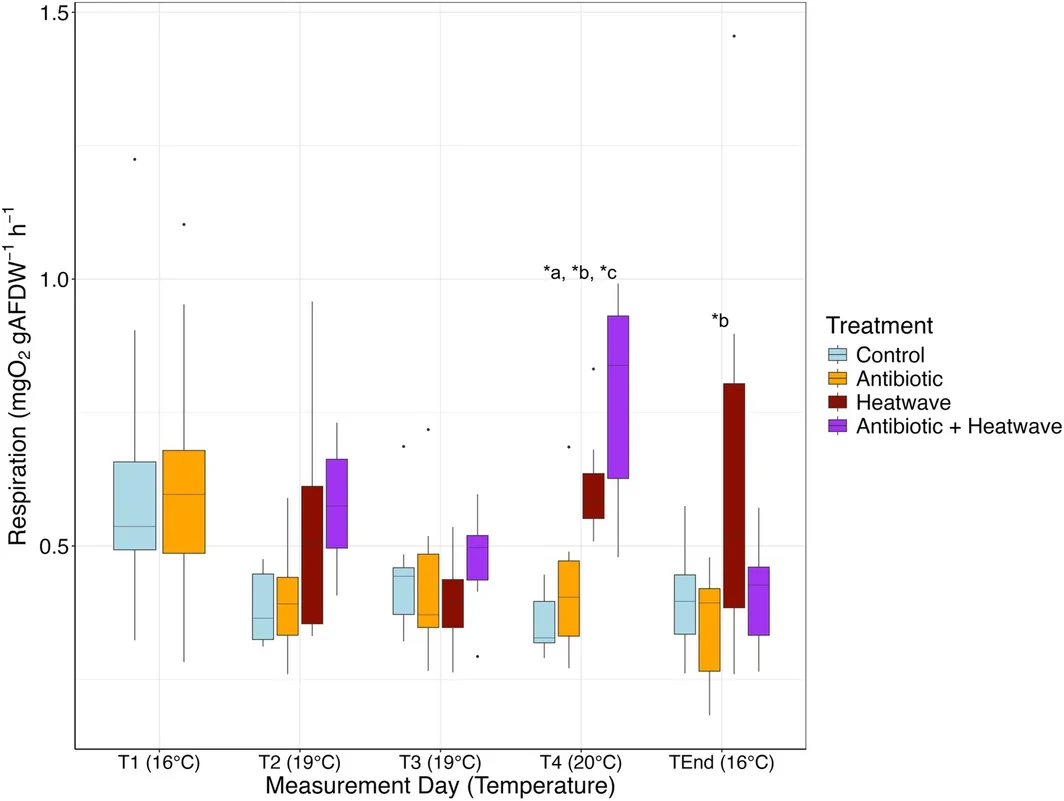
Median respiration rate (with 25th and 75th percentile, plus outliers) of sponges under each treatment at each time point throughout the experimental period. *a = control and heatwave treatments differ significantly, *b = control and antibiotic + heatwave treatments differ significantly, and *c = antibiotic and antibiotic + heatwave treatments differ significantly.

A linear mixed effects model showed that time, treatment, and the interactions between treatment and time all significantly affected sponge respiration rate (Table 4). Pairwise comparisons between time points showed that respiration rate did not differ significantly between any treatments at T1, T2, or T3 (Figure 6; Table S12). At T4, respiration rate of heatwave‐treated sponges was significantly higher than that of control sponges, and respiration rate of sponges under antibiotic + heatwave treatment was significantly higher than those of antibiotic‐treated sponges and control sponges (Figure 6; Table S12). Although respiration rates did not differ significantly between heatwave and antibiotic + heatwave treatments, the estimated difference in respiration between antibiotic + heatwave and control sponges was larger than that between heatwave and control sponges (Figure 6; Table S12). Interestingly at TEnd, antibiotic‐treated sponges had significantly lower respiration rates compared to heatwave‐treated sponges, despite respiration rates not differing between any other pairs of treatments (Figure 6; Table S12).

**TABLE 4:** Results from linear mixed effect models showing effects of time, treatment and their interaction on sponge respiration rate.

	DF	SS	MS	F value	*p* (BH)
Treatment	3	0.0967	0.0322	4.8709	0.0042**
Time	4	0.4155	0.1039	15.7008	0.0001**
Treatment × Time	10	0.2706	0.0271	4.0896	0.0001**

* *p* < 0.05.

** *p* < 0.01.

### 3.4 Changes in Specific Microbiome Members Correlate With Physiological Responses

Univariate tests identified 18 ASVs with read counts that significantly correlated with respiration rate. However, after Benjamini‐Hochberg correction, none of the correlations remained significant (Table S13), suggesting that the observed differences in community structure may be driven by multiple, modest changes across ASVs rather than a small number of strongly responsive ASVs. When ASV counts were aggregated at the family level, we identified eight families with read counts significantly correlated to respiration rate, all of which remained significant after *p*‐value correction. These families were *SOKP01, Akkermanciaceae*, and *UBA8477* (all negatively correlated with respiration rate), and *Rhodospirillaceae, Desulfobacteraceae*, *UBA2595*, *Alteromonadaceae*, and *Schleiferiaceae* (all positively correlated with respiration rate) (Table S14; Figure S8). Of these, *Akkermansiacea, Rhodospirillaceae, Alteromonadaceae* and *Schleiferiaceae* all differed significantly between treatments at least at one time point (Figure 4).

## 4 Discussion

This study aimed to disentangle the individual and combined effects of marine heatwaves (MHWs) and experimentally induced microbiome disruption on the physiological responses of the temperate sponge *Rowella lancifera*. Our results show that while antibiotic treatment and MHW conditions individually altered microbial diversity and community structure, their combined effects resulted in distinct physiological and microbial responses compared to either treatment alone. These findings suggest that microbial community composition may play a role in sponge physiological responses to thermal exposure, with implications for predicting sponge health and ecosystem functioning under future climate scenarios.

### 4.1 Antibiotic Treatment Causes Dysbiosis

Exposure to antibiotics caused significant changes to the microbial communities associated with *R. lancifera*, including reduced ASV richness, increased community dispersion, and shifts in overall microbial community structure. The increase in dispersion of microbial communities between antibiotic‐treated sponge individuals is consistent with the “Anna Karenina principle,” whereby healthy microbiomes tend to be similar, whilst those in a dysbiotic state exhibit greater variability and unique characteristics (Zaneveld et al. 2017). This is also supported by the decrease in ASV richness seen after antibiotic treatment, which is also seen the microbiomes of other marine invertebrates (Connelly et al. 2023; Griffin et al. 2021). Interestingly, ASV evenness increased as a result of antibiotic treatment, indicating a loss of dominant and potentially functionally important microbial taxa. This could open up ecological niches, allowing opportunistic microbes to colonise or increase in abundance (Webster and Taylor 2012; Gardères et al. 2015). The increases seen in the relative read abundance of certain microbial families including *Arcobacteraceae*, *Pelagibacteraceae*, *Flavobacteriaceae* and *Rhodobacteraceae*, some of which are known to contain disease‐causing genera (Buchan et al. 2005; Collado and Figueras 2011; Starliper 2011) immediately after antibiotic treatment could be evidence of an increase in opportunistic bacteria filling niches opened up by the initial effects of antibiotic treatment. This response supports previous findings that sponge‐associated microbiomes, although generally stable, can be sensitive to external disruption (Pita et al. 2018; Fan et al. 2012; Erwin et al. 2012; Morrow et al. 2016).

### 4.2 Marine Heatwaves Alone Cause Minor Changes to Native Microbiome

Unlike the antibiotic treatment, which increased community dispersion and caused immediate disruption to the sponge microbiome, heat exposure led to more structured microbiome changes that did not become apparent until the recovery period. The lesser impact of temperature on community structure when compared to antibiotic treatment suggests that the MHW treatment may have selectively impacted thermally sensitive taxa, rather than causing a random community collapse (Bell et al. 2024; Strano et al. 2023; de Castro‐Fernández et al. 2023). These taxon‐specific effects are also supported by the fact that many microbial families differed significantly between control and antibiotic‐treated sponges, yet only *Akkermansiaceae* significantly decreased under MHW conditions alone when compared to controls. Our results suggest that mild thermal exposure alone may not be sufficient to substantially alter the native microbiome of *R. lancifera*, which has also been observed in other sponge species (de Castro‐Fernández et al. 2023; Strand et al. 2017). In many sponge species, the microbial community remains stable across spatial and temporal gradients, even under changing environmental conditions (Thomas et al. 2016; Steinert et al. 2017; Glasl et al. 2018; Lamb and Watts 2023). However, as we are likely to see an increase in frequency and intensity of MHW events in the future (Oliver et al. 2018), and we did see some delayed responses in terms of shifts in microbial community structure after MHW exposure, the microbiome of *R. lancifera* may still be impacted under future ocean conditions.

### 4.3 Sponge Physiological Response During MHW and Microbial Dysbiosis

We found an increase in respiration rate in sponges under MHW conditions when compared to controls. Increased respiration rate has often been recorded in sponges under elevated temperature conditions, and as a result increased respiration rates are often used as a measure of physiological response to environmental change (Strano et al. 2022; Bennett et al. 2017; Ramsby et al. 2018). While respiration increases with temperature due to metabolic acceleration (Brown et al. 2004), elevated respiration under thermal exposure has been associated with physiological dysfunction in sponges when considered alongside other indicators. For example, increased oxygen consumption under elevated temperature has been linked to reduced biomass, altered cellular processes, and upregulation of stress‐related proteins, indicating disruption of cellular homeostasis (Bosch‐Belmar et al. 2023). Similarly, elevated temperatures can decouple metabolic demand from energy acquisition, with respiration remaining high while feeding rates decline, resulting in a negative energy balance that may impair long‐term survival (Beepat et al. 2021). Together, these findings suggest that increased respiration may reflect heightened energetic demand under thermal exposure and, when coupled with additional physiological changes, can be indicative of stress.

Interestingly, although antibiotic treatment did cause shifts in the sponges' microbial community structure, this effect alone did not induce responses in the form of increased respiration. This lack of response suggests that *R. lancifera* is able to manage short‐term shifts in microbial community structure without any detrimental effects. This has been seen in previous studies where microbiomes have shifted naturally in response to external stressors (Gastaldi et al. 2024; Vargas et al. 2021). Some sponge species show a degree of microbiome plasticity or functional redundancy, which may allow them to recover from microbial disruptions (Sacristán‐Soriano et al. 2020; Freeman et al. 2021). This redundancy can help maintain essential microbial functions, such as nutrient cycling, chemical defence and immune modulation, even if specific microbial taxa are lost (Pita et al. 2018; Gantt et al. 2019). Such plasticity could enhance sponge resilience by enabling microbial community restructuring in response to environmental change (Erwin et al. 2012; Björk et al. 2013).

Respiration rates increased under heatwave treatments, consistent with the well‐established temperature dependence of metabolic rates in ectotherms (Brown et al. 2004). While elevated respiration is often associated with physiological responses to environmental stressors in sponges (Strano et al. 2022; Bennett et al. 2017; Ramsby et al. 2018; Bosch‐Belmar et al. 2023; Beepat et al. 2021), increased respiration alone cannot distinguish between thermodynamic acceleration of metabolism and physiological stress. Notably, respiration rates did not differ significantly between heatwave and antibiotic + heatwave treatments, suggesting that temperature was the primary driver of the observed response. However, the larger estimated difference in respiration between control and antibiotic + heatwave‐treated sponges compared with control and heatwave‐treated sponges indicates that microbiome disruption may have influenced the magnitude of the physiological response to warming. Further work integrating additional stress markers would be needed to determine whether microbiome disruption alters sponge thermal tolerance.

### 4.4 Microbial Groups That Correlated With Physiological Response

Many families were impacted by antibiotic treatment, MHW exposure, or a combination of the two. However, the family *Akkermansiaceae* was the only to differ significantly between control and heatwave conditions alone, and was also one of the few families whose abundance was negatively correlated with respiration rate. Although microbes from the family *Akkermansiaceae* have not been functionally investigated in marine sponges, they are abundant in marine sediments and plankton blooms, and have recently been found a certain species of octocorals and kelps, where they were noted for their genetic potential for the degradation of sulphated methyl pentoses, dissimilatory nitrate and nitrite reduction, and urea hydrolysis (Orellana et al. 2022; Weigel et al. 2022; Monti et al. 2023). Interestingly, this family is well described in vertebrate guts, including some marine vertebrates, where they facilitate the breakdown of mucin (Cheutin et al. 2021; González et al. 2023). Although sponges do not produce mucins, they possess mucin‐like molecules that play roles in microbial management and waste expulsion (Lang et al. 2007; McGrath et al. 2017; Bakshani et al. 2018). If any of these important functions were impacted by a reduced abundance of microbes of the family *Akkermansiaceae*, then this could have negative consequences on the physiology of the sponge, including its respiration rate. Importantly, this family recovered after antibiotic treatment alone, and after MHW treatment alone, but was still significantly less abundant in sponges subjected to both treatments, highlighting the fact that dysbiosis has the potential to impact the resilience and recovery potential of *R. lancifera* to future MHW conditions.

The relative abundance of family *Rhodospirillaceae* also significantly (albeit positively) correlated with respiration rate and decreased in both antibiotic and antibiotic + heatwave treated sponges when compared to controls. *Rhodospirillaceae* are well described in sponge microbiomes and have the genetic capacity for aerobic sulfur oxidation and anaerobic denitrification (Karimi et al. 2018; Sugden et al. 2022). Increased respiration by the sponge will likely limit oxygen availability for the members of the family *Rhodospirillaceae*, which would then undertake heterotrophic denitrification over sulfur oxidation, which in turn would impact the S and N homeostasis in the microbiome. Further works is however required to understand how this could directly impact the sponge.

## 5 Conclusion

Our study suggests that the microbial community of the temperate sponge *R. lancifera* is associated with physiological responses to environmental change and is among the first studies to experimentally examine this relationship in a marine sponge. While both MHWs and antibiotic‐induced dysbiosis independently altered microbial community structure, temperature was the primary driver of changes in respiration rates. Although respiration rates did not differ significantly between heatwave and antibiotic + heatwave treatments at TEnd, the larger estimated difference in respiration between control and antibiotic + heatwave‐treated sponges at T4 suggests that microbiome disruption may influence short‐term physiological responses to warming. These findings highlight the potential importance of microbial‐mediated functions in maintaining host homeostasis and suggest that future increases in the frequency of anthropogenic stressors may affect sponge health and ecosystem functioning through microbial community disruption. There are likely to be cases in the future where dysbiosis occurs naturally in sponges as a result of direct and indirect anthropogenic impacts, including ocean acidification (Ribes et al. 1999; Botté et al. 2019), pollution (Gastaldi et al. 2024; Vad et al. 2022), and warming (Bell et al. 2024; Strano et al. 2023; Vargas et al. 2021), potentially altering sponge responses to additional stressors such as MHWs. As climate change continues to intensify, understanding the role of sponge‐associated microbial communities may be important for predicting the resilience of benthic marine ecosystems.

## Author Contributions

**James J. Bell:** conceptualization, funding acquisition, writing – review and editing, methodology, supervision. **Alice Rogers:** writing – review and editing, supervision. **Torsten Thomas:** conceptualization, writing – review and editing, methodology. **Manon Broadribb:** conceptualization, investigation, writing – original draft, methodology, writing – review and editing, formal analysis, data curation.

## Funding

This work was supported by Victoria University of Wellington and George Mason Charitable Trust.

## Ethics Statement

The authors have nothing to report.

## Consent

The authors have nothing to report.

## Conflicts of Interest

The authors declare no conflicts of interest.

## Supporting information


**Figure S1:** Map of New Zealand and the Fiordland Marine Area showing the position of our sponge collection site in Bradshaw Sound, Doubtful Sound.
**Figure S2:** Schematic representation of experimental set up with flow through and temperature control systems.
**Figure S3:** Daily mean SST (black line) based on NOAA OISST data from the 1/4° area closest to my sampling sites between May 2020 and March 2023. The black dashed line indicates the climatological mean (calculated over the 1st January 1993 to 1st June 2023), the orange dashed line marks the 90th percentile threshold for a moderate MHW, and the red dashed line marks the strong MHW threshold, defined as the 90th percentile plus the difference between the climatological mean and the 90th percentile (Hobday et al. 2018).
**Figure S4:** Regression of sponge ash‐free dry weight against buoyant weight, used to calculate ash‐free dry weight from buoyant weight measurements taken throughout the experiment.
**Figure S5:** Rarefaction curves representing the number of OTUs obtained for each of the sponges at T1, T4 and TEnd under each treatment.
**Figure S6:** Median distance (with 5th, 25th, 75th and 95th percentile, plus outliers) of microbial community samples to group centroids under each treatment at each time point.
**Figure S7:** Non‐metric multidimensional scaling (nMDS) plot of microbial communities at different time points under (A) control conditions, (B) heatwave treatment, (C) antibiotic treatment, and (D) antibiotic + heatwave treatment. Large points represent group centroids, small points represent individual samples, and ellipses represent 95% confidence around the centroid. NB at T0 all treatment tanks were under control conditions, and at T1 only antibiotic treatment had been administered. By T4 both antibiotic treatment and heat stress had been administered.
**Figure S8:** Scatter plots with trendlines showing the relationship between respiration rate and total read count of microbial families. Families plotted are those where the relationship was significant.
**Table S1:** Calculation of average time for *R. lancifera* to pump 4 L of water (i.e., one full treatment tank) to inform antibiotic incubation time without flow. Flow rate = oscula area × excurrent velocity × 0.0036 to give L/h. Hours before change = volume of tank/flow rate.
**Table S2:** Experimental phases by day and date, including temperature regime for heatwave and heatwave + antibiotic treated sponges. Control sponges and antibiotic treated sponges remained at 16°C throughout the experiment.
**Table S3:** Main results from PERMANOVA tests to show effect of treatment on microbial community structure at different time points throughout the experimental period. * = *p* < 0.05, ** = *p* < 0.01.
**Table S4:** Results from PERMDISP analysis showing differences in dispersion of microbial communities from their group centroids between treatments at T4 and TEnd. * = *p* < 0.05, ** = *p* < 0.01.
**Table S5:** Post hoc pairwise analyses of PERMANOVA results showing differences in microbial communities between time points under each treatment. * = *p* < 0.05, ** = *p* < 0.01.
**Table S6:** Table of results from post hoc pairwise analyses of differences in mean ASV richness between microbial communities of sponges under different treatments at each time point.* = *p* < 0.05, ** = *p* < 0.01.
**Table S7:** Table of results from post hoc pairwise analyses of differences in mean Pielou's evenness microbial communities of sponges under different treatments at each time point. * = *p* < 0.05, ** = *p* < 0.01.
**Table S8:** Table of results from post hoc pairwise analyses of differences in mean Shannon diversity between microbial communities of sponges under different treatments at each time point. * = *p* < 0.05, ** = *p* < 0.01.
**Table S9:** Results from ANCOMBC analysis showing differentially abundant microbial families between control and antibiotic treated sponges at T1. Log fold changes are on the natural log scale and indicate differences in antibiotic treated sponges when compared to control sponges. * = *p* < 0.05, ** = *p* < 0.01.
**Table S10:** Results from ANCOMBC analysis showing differentially abundant microbial families between all treatments at T4. Log fold changes are on the natural log scale and indicate differences in antibiotic treated sponges when compared to control sponges. * = *p* < 0.05, ** = *p* < 0.01.
**Table S11:** Results from ANCOMBC analysis showing differentially abundant microbial families between treatments at TEnd. Log fold changes are on the natural log scale and indicate differences in antibiotic treated sponges when compared to control sponges. * = *p* < 0.05, ** = *p* < 0.01.
**Table S12:** Table of results from post hoc pairwise analyses of differences in mean respiration rate between sponges under different treatments at each time point. * = *p* < 0.05, ** = *p* < 0.01.
**Table S13:** Results from multivariate GLM showing correlations between individual ASV read counts and respiration rate. ASVs listed are those which were significantly correlated with respiration rate before *p*‐value correction. * = *p* < 0.05, ** = *p* < 0.01.
**Table S14:** Results from multivariate GLM showing correlations between individual family read counts and respiration rate. Families listed are those which were significantly correlated with respiration rate before *p*‐value correction. * = *p* < 0.05, ** = *p* < 0.01.

## Data Availability

The datasets generated and/or analysed during the current study, along with the R scripts used for data analysis, are available in the figshare repository, doi: 10.6084/m9.figshare.30204412. The raw 16S rRNA gene amplicon sequencing data generated in this study have been deposited in the NCBI Sequence Read Archive (SRA) under BioSample accession numbers SAMN52852118–SAMN52852212, as part of BioProject PRJNA1347189. Associated metadata are available within the BioSample records and in the Supporting Information accompanying this article.
