# Supplementary material for: Induced Dysbiosis Modifies Physiological Responses to Warming in a Temperate Sponge
Source: Environ Microbiol. 2026 Sep 6;28(9):e70402. doi: 10.1111/1462-2920.70402 (PMC13547887; doi:10.1111/1462-2920.70402)
Supplement: Supplementary file 1 [file EMI-28-e70402-s001.docx]

Supplementary Information

Figure S1: Map of New Zealand and the Fiordland Marine Area showing the position of our sponge collection site in Bradshaw Sound, Doubtful Sound

Figure S2: Schematic representation of experimental set up with flow through and temperature control systems.

Figure S3: Daily mean SST (black line) based on NOAA OISST data from the 1/4° area closest to my sampling sites between May 2020 and March 2023. The black dashed line indicates the climatological mean (calculated over the 1^st^ January 1993 to 1^st^ June 2023), the orange dashed line marks the 90th percentile threshold for a moderate MHW, and the red dashed line marks the strong MHW threshold, defined as the 90th percentile plus the difference between the climatological mean and the 90th percentile (Hobday et al., 2018)

Figure S4: Regression of sponge ash-free dry weight against buoyant weight, used to calculate ash-free dry weight from buoyant weight measurements taken throughout the experiment.

Figure S5: Rarefaction curves representing the number of OTUs obtained for each of the sponges at T1, T4 and TEnd under each treatment.

Figure S6: Median distance (with 5^th^, 25^th^, 75^th^ and 95^th^ percentile, plus outliers) of microbial community samples to group centroids under each treatment at each time point.

Figure S7: Non-metric multidimensional scaling (nMDS) plot of microbial communities at different time points under A) control conditions, B) heatwave treatment, C) antibiotic treatment, and D) antibiotic + heatwave treatment. Large points represent group centroids, small points represent individual samples, and ellipses represent 95% confidence around the centroid. NB at T0 all treatment tanks were under control conditions, and at T1 only antibiotic treatment had been administered. By T4 both antibiotic treatment and heat stress had been administered.

Figure S8: Scatter plots with trendlines showing the relationship between respiration rate and total read count of microbial families. Families plotted are those where the relationship was significant.

Table S1: Calculation of average time for *R. lancifera* to pump 4L of water (i.e. one full treatment tank) to inform antibiotic incubation time without flow. Flow rate = oscula area x excurrent velocity x 0.0036 to give L/h. Hours before change = volume of tank / flow rate.

| Sponge | Repeat | Osculum Area (mm2) | Velocity (mm/s) | Flow Rate (L/h) | Hours Before Change | Average |
| --- | --- | --- | --- | --- | --- | --- |
| C2.1 | 1 | 12.566 | 5 | 0.226 | 17.6991 | 12.12556 |
| C2.1 | 2 | 12.566 | 10 | 0.452 | 8.84956 |  |
| C2.1 | 3 | 12.566 | 9 | 0.407 | 9.82801 |  |
| T1.3.1 | 1 | 9.621 | 4 | 0.139 | 28.7770 | 35.23335 |
| T1.3.1 | 2 | 9.621 | 3 | 0.104 | 38.4615 |  |
| T1.3.1 | 3 | 9.621 | 4 | 0.104 | 38.4615 |  |
| T2.3.2 | 1 | 7.069 | 8 | 0.204 | 19.6078 | 22.74118 |
| T2.3.2 | 2 | 7.069 | 7 | 0.178 | 22.4719 |  |
| T2.3.2 | 3 | 7.069 | 6 | 0.153 | 26.1438 |  |
| T3.3.1 | 1 | 15.904 | 10 | 0.573 | 6.98080 | 14.74087 |
| T3.3.1 | 2 | 15.904 | 5 | 0.286 | 13.9860 |  |
| T3.3.1 | 3 | 15.904 | 3 | 0.172 | 23.2558 |  |
| T2.1.2 | 1 | 13.1416 | 3 | 0.142 | 28.1690 | 23.49901 |
| T2.1.2 | 2 | 13.1416 | 4 | 0.189 | 21.1640 |  |
| T2.1.2 | 3 | 13.1416 | 4 | 0.189 | 21.1640 |  |

Table S2: Experimental phases by day and date, including temperature regime for heatwave and heatwave + antibiotic treated sponges. Control sponges and antibiotic treated sponges remained at 16°C throughout the experiment.

| Day | Date | Phase | Temp (MHW, MHW + AB) |
| --- | --- | --- | --- |
| Day 1 | 15.01.2024 | Acclimation | Ambient (16°C) |
| Day 2 | 16.01.2024 | Acclimation | Ambient (16°C) |
| Day 3 | 17.01.2024 | Acclimation | Ambient (16°C) |
| Day 4 | 18.01.2024 | Acclimation | Ambient (16°C) |
| Day 5 | 19.01.2024 | Acclimation | Ambient (16°C) |
| Day 6 | 20.01.2024 | Acclimation | Ambient (16°C) |
| Day 7 | 21.01.2024 | Acclimation | Ambient (16°C) |
| Day 8 | 22.01.2024 | Acclimation | Ambient (16°C) |
| Day 9 | 23.01.2024 | Acclimation | Ambient (16°C) |
| Day 10 | 24.01.2024 | Acclimation | Ambient (16°C) |
| Day 11 | 25.01.2024 | Acclimation | Ambient (16°C) |
| Day 12 | 26.01.2024 | Acclimation | Ambient (16°C) |
| Day 13 | 27.01.2024 | Acclimation | Ambient (16°C) |
| Day 14 | 28.01.2024 | Acclimation | Ambient (16°C) |
| Day 15 | 29.01.2024 | Resp, BW and tissue sample T0 | Ambient (16°C) |
| Day 16 | 30.01.2024 | AB treatment | Ambient (16°C) |
| Day 17 | 31.01.2024 | AB treatment | Ambient (16°C) |
| Day 18 | 01.02.2024 | AB treatment | Ambient (16°C) |
| Day 19 | 02.02.2024 | Resp, BW and tissue sample T1 | Ambient (16°C) |
| Day 20 | 03.02.2024 | MHW ramp up | Ambient + 1 (17°C) |
| Day 21 | 04.02.2024 | MHW ramp up | Ambient + 2 (18°C) |
| Day 22 | 05.02.2024 | MHW ramp up | Ambient + 3 (19°C) |
| Day 23 | 06.02.2024 | Resp and BW T2 | Ambient + 3 (19°C) |
| Day 24 | 07.02.2024 | MHW peak | Ambient + 3 (19°C) |
| Day 25 | 08.02.2024 | MHW peak | Ambient + 3 (19°C) |
| Day 26 | 09.02.2024 | MHW peak | Ambient + 3 (19°C) |
| Day 27 | 10.02.2024 | Resp and BW T3 | Ambient + 3 (19°C) |
| Day 28 | 11.02.2024 | MHW peak | Ambient + 4 (20°C) |
| Day 29 | 12.02.2024 | MHW peak | Ambient + 4 (20°C) |
| Day 30 | 13.02.2024 | MHW peak | Ambient + 4 (20°C) |
| Day 31 | 14.02.2024 | Resp, BW and tissue sample T4 | Ambient + 4 (20°C) |
| Day 32 | 15.02.2024 | Recovery | Ambient + 3 (19°C) |
| Day 33 | 16.02.2024 | Recovery | Ambient +2 (18°C) |
| Day 34 | 17.02.2024 | Recovery | Ambient +1 (17°C) |
| Day 35 | 18.02.2024 | Recovery | Ambient (16°C) |
| Day 36 | 19.02.2024 | Recovery | Ambient (16°C) |
| Day 37 | 20.02.2024 | Recovery | Ambient (16°C) |
| Day 38 | 21.02.2024 | Resp, BW and tissue sample TEnd | Ambient (16°C) |
| Day 40 | 22.02.2024 | Weigh and dry sponges | NA |

Table S3: Main results from PERMANOVA tests to show effect of treatment on microbial community structure at different time points throughout the experimental period. * = p < 0.05, ** = p < 0.01.

| T0 | DF | SS | MS | F Value | R^2^ | P Value |
| --- | --- | --- | --- | --- | --- | --- |
| Treatment | 3 | 0.1607 | 0.0536 | 1.1436 | 0.1464 | 0.2469 |
| Residuals | 20 | 0.9368 | 0.0468 |  | 0.8536 |  |
| Total | 23 | 1.0976 |  |  | 1 |  |
| T1 | **DF** | **SS** | **MS** | **F Value** | **R^2^** | **P Value** |
| Treatment | 1 | 0.2249 | 0.2249 | 3.3855 | 0.1388 | 0.0001** |
| Residuals | 21 | 1.3953 | 0.0664 |  | 0.8612 |  |
| Total | 22 | 1.6202 |  |  | 1 |  |
| T4 | **DF** | **SS** | **MS** | **F Value** | **R^2^** | **P Value** |
| Treatment | 3 | 0.8971 | 0.2990 | 3.0434 | 0.3134 | 0.0007** |
| Residuals | 20 | 1.9651 | 0.0983 |  | 0.6866 |  |
| Total | 23 | 2.8622 |  |  | 1 |  |
| TEnd | **DF** | **SS** | **MS** | **F Value** | **R^2^** | **P Value** |
| Treatment | 3 | 1.0934 | 0.3645 | 3.5146 | 0.3452 | 0.0001** |
| Residuals | 20 | 2.0739 | 0.1037 |  | 0.6548 |  |
| Total | 23 | 3.1673 |  |  | 1.0000 |  |

Table S4: Results from PERMDISP analysis showing differences in dispersion of microbial communities from their group centroids between treatments at T4 and TEnd. * = p < 0.05, ** = p < 0.01.

| Time | Pairs | P (BH) |
| --- | --- | --- |
| T1 | Control-Antibiotic | 0.0003** |
| T4 | Control-Heatwave | 0.4706 |
|  | Control-Antibiotic | 0.2166 |
|  | Control-Antibiotic + Heatwave | 0.2217 |
|  | Heatwave-Antibiotic | 0.4552 |
|  | Heatwave-Antibiotic + Heatwave | 0.5210 |
|  | Antibiotic-Antibiotic + Heatwave | 0.5210 |
| TEnd | Control-Heatwave | 0.3280 |
|  | Control-Antibiotic | 0.3280 |
|  | Control-Antibiotic + Heatwave | 0.0936 |
|  | Heatwave-Antibiotic | 0.9267 |
|  | Heatwave-Antibiotic + Heatwave | 0.6426 |
|  | Antibiotic-Antibiotic + Heatwave | 0.6380 |

Table S5: Post-hoc pairwise analyses of PERMANOVA results showing differences in microbial communities between time points under each treatment. * = p < 0.05, ** = p < 0.01.

| Treatment | Pairs | DF | SS | F value | R^2^ | P (BH) |
| --- | --- | --- | --- | --- | --- | --- |
| Control | T0 vs T1 | 1 | 0.0201 | 0.7993 | 0.0740 | 0.5277 |
|  | T0 vs T4 | 1 | 0.0426 | 1.4002 | 0.1228 | 0.3549 |
|  | T0 vs TEnd | 1 | 0.1130 | 6.7552 | 0.4032 | 0.0144* |
|  | T1 vs T4 | 1 | 0.0318 | 0.8807 | 0.0809 | 0.5277 |
|  | T1 vs TEnd | 1 | 0.1450 | 6.4591 | 0.3924 | 0.0189* |
|  | T4 vs TEnd | 1 | 0.0800 | 2.8889 | 0.2241 | 0.1214 |
| Heatwave | T0 vs T1 | 1 | 0.0490 | 1.7969 | 0.1523 | 0.1624 |
|  | T0 vs T4 | 1 | 0.3060 | 13.3527 | 0.5718 | 0.0069** |
|  | T0 vs TEnd | 1 | 0.1183 | 1.7413 | 0.1483 | 0.1624 |
|  | T1 vs T4 | 1 | 0.2292 | 8.5477 | 0.4608 | 0.0069** |
|  | T1 vs TEnd | 1 | 0.0611 | 0.8503 | 0.0784 | 0.5257 |
|  | T4 vs TEnd | 1 | 0.1571 | 2.3282 | 0.1889 | 0.1186 |
| Antibiotic | T0 vs T1 | 1 | 0.0346 | 0.4669 | 0.0493 | 0.6886 |
|  | T0 vs T4 | 1 | 0.5908 | 5.0373 | 0.3350 | 0.0366* |
|  | T0 vs TEnd | 1 | 0.4259 | 8.4495 | 0.4580 | 0.0216* |
|  | T1 vs T4 | 1 | 0.3844 | 2.7183 | 0.2320 | 0.1598 |
|  | T1 vs TEnd | 1 | 0.2281 | 3.3985 | 0.2741 | 0.0930 |
|  | T4 vs TEnd | 1 | 0.1703 | 1.5336 | 0.1330 | 0.2952 |
| Antibiotic + Heatwave | T0 vs T1 | 1 | 0.0389 | 0.5975 | 0.0564 | 0.5477 |
|  | T0 vs T4 | 1 | 0.2663 | 3.8950 | 0.2803 | 0.0447* |
|  | T0 vs TEnd | 1 | 0.3920 | 8.0085 | 0.4447 | 0.0108* |
|  | T1 vs T4 | 1 | 0.2159 | 3.5571 | 0.2624 | 0.0447* |
|  | T1 vs TEnd | 1 | 0.3527 | 8.5470 | 0.4608 | 0.0108* |
|  | T4 vs TEnd | 1 | 0.0529 | 1.1875 | 0.1061 | 0.3518 |

Table S6: Table of results from post-hoc pairwise analyses of differences in mean ASV richness between microbial communities of sponges under different treatments at each time point.* = p < 0.05, ** = p < 0.01.

| Time | Contrast | Estimate | SE | DF | T Ratio | P (BH) |
| --- | --- | --- | --- | --- | --- | --- |
| T1 | Control vs. Antibiotic | -3.1117 | 0.9054 | 23.5789 | -3.4368 | 0.0095** |
| T4 | Control vs. Heatwave | 0.8318 | 1.2089 | 47.0733 | 0.6881 | 0.5360 |
|  | Control vs. Antibiotic | -3.4552 | 1.2347 | 33.1368 | -2.7985 | 0.0276* |
|  | Control vs. (Antibiotic + Heatwave) | -1.9686 | 1.2347 | 33.1368 | -1.5945 | 0.1955 |
|  | Heatwave vs. Antibiotic | -4.2870 | 1.2347 | 33.1368 | -3.4722 | 0.0095** |
|  | Heatwave vs. (Antibiotic + Heatwave) | -2.8004 | 1.2347 | 33.1368 | -2.2682 | 0.0556 |
|  | Antibiotic vs. (Antibiotic + Heatwave) | 1.4866 | 1.2166 | 45.9712 | 1.2219 | 0.3060 |
| TEnd | Control vs. Heatwave | -3.0401 | 1.2089 | 47.0733 | -2.5148 | 0.0333* |
|  | Control vs. Antibiotic | -3.2479 | 1.2347 | 33.1368 | -2.6306 | 0.0333* |
|  | Control - (Antibiotic + Heatwave) | -4.5323 | 1.2347 | 33.1368 | -3.6709 | 0.0095** |
|  | Heatwave vs. Antibiotic | -0.2078 | 1.2347 | 33.1368 | -0.1683 | 0.8674 |
|  | Heatwave vs. (Antibiotic + Heatwave) | -1.4923 | 1.2347 | 33.1368 | -1.2087 | 0.3060 |
|  | Antibiotic vs. (Antibiotic + Heatwave) | -1.2845 | 1.2166 | 45.9712 | -1.0558 | 0.3505 |

Table S7: Table of results from post-hoc pairwise analyses of differences in mean Pielou’s evenness microbial communities of sponges under different treatments at each time point. * = p < 0.05, ** = p < 0.01.

| Time | Contrast | Estimate | SE | DF | T Ratio | P (BH) |
| --- | --- | --- | --- | --- | --- | --- |
| T1 | Control vs. Antibiotic | 0.0321 | 0.0217 | 42.1904 | 1.4785 | 0.1907 |
| T4 | Control vs. Heatwave | 0.0739 | 0.0310 | 41.0895 | 2.3866 | 0.0353* |
|  | Control vs. Antibiotic | 0.1821 | 0.0305 | 41.0895 | 5.9787 | 0.0001** |
|  | Control vs. (Antibiotic + Heatwave) | 0.1400 | 0.0305 | 41.0895 | 4.5959 | 0.0003** |
|  | Heatwave vs. Antibiotic | 0.1081 | 0.0305 | 41.0895 | 3.5512 | 0.0018** |
|  | Heatwave vs. (Antibiotic + Heatwave) | 0.0660 | 0.0305 | 41.0895 | 2.1685 | 0.0519 |
|  | Antibiotic vs. (Antibiotic + Heatwave) | -0.0421 | 0.0309 | 41.0895 | -1.3634 | 0.2129 |
| TEnd | Control vs. Heatwave | -0.0114 | 0.0310 | 41.0895 | -0.3681 | 0.7742 |
|  | Control vs. Antibiotic | 0.1105 | 0.0305 | 41.0895 | 3.7933 | 0.0011** |
|  | Control vs. (Antibiotic + Heatwave) | 0.1101 | 0.0305 | 41.0895 | 3.7801 | 0.0011** |
|  | Heatwave vs. Antibiotic | 0.1269 | 0.0305 | 41.0895 | 4.1677 | 0.0005** |
|  | Heatwave - (Antibiotic + Heatwave) | 0.1265 | 0.0305 | 41.0895 | 4.1545 | 0.0005** |
|  | Antibiotic - (Antibiotic + Heatwave) | -0.0004 | 0.0309 | 41.0895 | -0.0131 | 0.9896 |

Table S8: Table of results from post-hoc pairwise analyses of differences in mean Shannon diversity between microbial communities of sponges under different treatments at each time point. * = p < 0.05, ** = p < 0.01.

| Time | Contrast | Estimate | SE | DF | T Ratio | P (BH) |
| --- | --- | --- | --- | --- | --- | --- |
| T1 | Control vs. Antibiotic | -0.0261 | 0.0478 | 39.2262 | -0.5462 | 0.6070 |
| T4 | Control vs. Heatwave | 0.1872 | 0.0683 | 41.4499 | 2.7415 | 0.0234* |
|  | Control vs. Antibiotic | 0.3054 | 0.0671 | 39.6911 | 4.5538 | 0.0006** |
|  | Control vs. (Antibiotic + Heatwave) | 0.2330 | 0.0671 | 39.6911 | 3.4739 | 0.0041** |
|  | Heatwave vs. Antibiotic | 0.1183 | 0.0671 | 39.6911 | 1.7633 | 0.1240 |
|  | Heatwave vs. (Antibiotic + Heatwave) | 0.0458 | 0.0671 | 39.6911 | 0.6834 | 0.5889 |
|  | Antibiotic vs. (Antibiotic + Heatwave) | -0.0724 | 0.0681 | 41.1584 | -1.0639 | 0.3816 |
| TEnd | Control vs. Heatwave | -0.1256 | 0.0683 | 41.4499 | -1.8402 | 0.1240 |
|  | Control vs. Antibiotic | 0.1534 | 0.0671 | 39.6911 | 2.2877 | 0.0597 |
|  | Control vs. (Antibiotic + Heatwave) | 0.1181 | 0.0671 | 39.6911 | 1.7615 | 0.1240 |
|  | Heatwave vs. Antibiotic | 0.2791 | 0.0671 | 39.6911 | 4.1608 | 0.0011** |
|  | Heatwave vs. (Antibiotic + Heatwave) | 0.2438 | 0.0671 | 39.6911 | 3.6346 | 0.0034** |
|  | Antibiotic vs. (Antibiotic + Heatwave) | -0.0353 | 0.0681 | 41.1584 | -0.5184 | 0.6070 |

Table S9: Results from ANCOMBC analysis showing differentially abundant microbial families between control and antibiotic treated sponges at T1. Log fold changes are on the natural log scale and indicate differences in antibiotic treated sponges when compared to control sponges. . * = p < 0.05, ** = p < 0.01.

| Family | Log_2_ Fold  Changes | P (BH) | CTRL total read count | AB total read count |
| --- | --- | --- | --- | --- |
| *Arcobacteraceae* | 3.6452 | **0.0022**** | 63 | 623 |
| *UBA868* | 2.7151 | **0.0022**** | 666 | 3809 |
| *Flavobacteriaceae* | 2.6774 | **0.0022**** | 2101 | 14014 |
| *Micavibrionaceae* | 2.5870 | **0.0022**** | 53 | 318 |
| *Pelagibacteraceae* | 2.9021 | **0.0022**** | 488 | 1912 |
| *Methylophilaceae* | 2.6263 | **0.0022**** | 3680 | 19697 |
| *Alteromonadaceae* | 2.7109 | **0.0022**** | 32 | 214 |
| *UBA2595* | 2.3676 | **0.0029**** | 119 | 413 |
| *Cellvibrionaceae* | 3.0281 | **0.0029**** | 228 | 1366 |
| *Rhodobacteraceae* | 2.3527 | **0.0033**** | 928 | 3630 |
| *4be13* | 3.3466 | **0.0055**** | 90 | 886 |
| *Schleiferiaceae* | 2.3387 | **0.0062**** | 5462 | 28783 |
| *Thioglobaceae* | 2.1990 | **0.0067**** | 55 | 141 |
| *Pirellulaceae* | 2.2612 | **0.0108*** | 123 | 522 |
| *Cyanobiaceae* | 2.4646 | **0.0108*** | 250 | 748 |
| *D2472* | 2.1180 | **0.0113*** | 34 | 282 |
| *Puniceispirillaceae* | 2.2295 | **0.0113*** | 78 | 229 |
| *DEV007* | 2.3472 | **0.0113*** | 197 | 1329 |
| *Enterobacteriaceae* | 2.1494 | **0.0172*** | 199 | 569 |
| *Nitrincolaceae* | 2.4228 | **0.0249*** | 83 | 486 |
| *Vibrionaceae* | 1.8591 | **0.0249*** | 65 | 299 |
| *Mycobacteriaceae* | -1.9614 | **0.0303*** | 1480 | 357 |
| *Aestuariivirgaceae* | 1.9301 | **0.0303*** | 183 | 562 |
| *Mycobacteriaceae* | 1.5454 | **0.0303*** | 117 | 260 |
| *Planctomycetaceae* | 1.7773 | 0.0742 | 140 | 360 |
| *Ketobacteraceae* | 3.6452 | 0.0589 | 63 | 623 |

Table S10: Results from ANCOMBC analysis showing differentially abundant microbial families between all treatments at T4. Log fold changes are on the natural log scale and indicate differences in antibiotic treated sponges when compared to control sponges. . * = p < 0.05, ** = p < 0.01.

|  | **Log_2_ fold changes** | | | | | | **BH corrected p-value** | | | | | | **Total ASV read count** | | | |
| --- | --- | --- | --- | --- | --- | --- | --- | --- | --- | --- | --- | --- | --- | --- | --- | --- |
| **Family** | **CTRL-**  **HW** | **CTRL-**  **AB** | **CTRL-**  **AH** | **HW-**  **AB** | **HW-**  **AH** | **AB-**  **AH** | **CTRL-**  **HW** | **CTRL-**  **AB** | **CTRL-**  **AH** | **HW-**  **AB** | **HW-**  **AH** | **AB-**  **AH** | **CTRL** | **HW** | **AB** | **AH** |
| **Rhodospirillaceae** | 0.25 | -8.53 | -9.81 | -8.79 | -10.06 | -1.28 | 1.000 | **0.001**** | **0.001**** | **0.001**** | **0.005**** | 1.000 | 97143 | 68632 | 865 | 22437 |
| **NAC60-12** | -0.20 | -9.53 | -9.04 | -9.34 | -8.84 | 0.49 | 1.000 | **0.001**** | **0.006**** | **0.006**** | **0.029*** | 1.000 | 135518 | 88085 | 3085 | 35962 |
| **Aestuariivirgaceae** | 0.27 | -5.25 | -5.88 | -5.52 | -6.15 | -0.63 | 1.000 | **0.001**** | **0.001**** | **0.001**** | **0.001**** | 1.000 | 558 | 270 | 8 | 11 |
| **Thiohalobacteraceae** | -0.13 | -3.61 | -3.95 | -3.48 | -3.82 | -0.34 | 1.000 | **0.001**** | **0.004**** | **0.013*** | **0.020*** | 1.000 | 15204 | 3314 | 944 | 1840 |
| **UBA2165** | -0.53 | -5.81 | -6.99 | -5.28 | -6.46 | -1.18 | 1.000 | **0.001**** | **0.017*** | **0.035*** | 0.065 | 1.000 | 8909 | 5697 | 230 | 1636 |
| **Pseudohongiellaceae** | -0.40 | -5.29 | -5.09 | -4.90 | -4.69 | 0.21 | 1.000 | **0.002**** | **0.007**** | **0.014*** | **0.032*** | 1.000 | 919 | 245 | 32 | 157 |
| **Sphingomonadaceae** | -0.95 | -5.84 | -3.01 | -4.90 | -2.06 | 2.83 | 1.000 | **0.015*** | 1.000 | **0.020*** | 1.000 | 1.000 | 2662 | 297 | 45 | 1769 |
| **Akkermansiaceae** | -4.77 | -4.06 | -5.80 | 0.71 | -1.03 | -1.75 | **0.002**** | **0.021*** | **0.001**** | 1.000 | 1.000 | 1.000 | 2200 | 34 | 229 | 83 |
| **Endozoicomonadaceae** | -1.24 | -2.73 | -3.67 | -1.49 | -2.43 | -0.94 | 1.000 | **0.037*** | 0.112 | 1.000 | 1.000 | 1.000 | 1280 | 351 | 314 | 629 |
| **Lacipirellulaceae** | 2.94 | 2.37 | 1.41 | -0.57 | -1.53 | -0.96 | 0.059 | 0.098 | 1.000 | 1.000 | 1.000 | 1.000 | 9 | 15 | 64 | 104 |
| **Halieaceae** | 2.25 | 2.32 | 0.64 | 0.07 | -1.61 | -1.67 | 0.731 | 0.226 | 1.000 | 1.000 | 1.000 | 1.000 | 9 | 21 | 61 | 29 |
| **GLO-3** | -0.61 | -3.28 | -4.69 | -2.68 | -4.09 | -1.41 | 1.000 | 0.268 | **0.035*** | 1.000 | 0.459 | 1.000 | 31644 | 17772 | 7268 | 5954 |
| **Alteromonadaceae** | 0.86 | 1.88 | 0.33 | 1.02 | -0.53 | -1.55 | 1.000 | 0.310 | 1.000 | 1.000 | 1.000 | 1.000 | 85 | 42 | 488 | 222 |
| **Pirellulaceae** | 2.72 | 1.89 | 1.73 | -0.84 | -0.99 | -0.16 | 0.191 | 0.295 | 1.000 | 1.000 | 1.000 | 1.000 | 69 | 166 | 216 | 406 |
| **Micavibrionaceae** | 1.83 | 2.07 | 1.85 | 0.25 | 0.02 | -0.23 | 0.712 | 0.372 | 1.000 | 1.000 | 1.000 | 1.000 | 11 | 9 | 48 | 116 |
| **DEV007** | 1.54 | 1.49 | 1.37 | -0.05 | -0.17 | -0.11 | 0.984 | 0.456 | 1.000 | 1.000 | 1.000 | 1.000 | 96 | 76 | 262 | 767 |
| **Flavobacteriaceae** | 2.38 | 1.98 | 1.81 | -0.40 | -0.57 | -0.17 | 0.197 | 0.446 | 0.505 | 1.000 | 1.000 | 1.000 | 57 | 61 | 392 | 309 |
| **Tenderiaceae** | -0.11 | -1.57 | -3.90 | -1.45 | -3.79 | -2.33 | 1.000 | 0.543 | 0.072 | 1.000 | 0.188 | 0.785 | 354 | 118 | 134 | 114 |
| **Nitrospinaceae** | 2.36 | -4.89 | -2.36 | -7.25 | -4.72 | 2.53 | 1.000 | 0.620 | 1.000 | **0.044*** | 0.057 | 1.000 | 100493 | 65888 | 8228 | 11000 |
| **Coxiellaceae** | 1.94 | 1.65 | 0.83 | -0.29 | -1.11 | -0.82 | 0.518 | 0.963 | 1.000 | 1.000 | 1.000 | 1.000 | 7 | 4 | 42 | 31 |
| **Natronospirillaceae** | 2.65 | 1.39 | 0.11 | -1.25 | -2.54 | -1.28 | 0.308 | 1.000 | 1.000 | 1.000 | 0.657 | 1.000 | 17 | 19 | 44 | 30 |
| **Francisellaceae** | 2.14 | -3.17 | -0.99 | -5.31 | -3.12 | 2.18 | 0.142 | 1.000 | 1.000 | 0.209 | 0.117 | 1.000 | 503580 | 605231 | 293520 | 498906 |
| **Nitrosopumilaceae** | 3.06 | 1.23 | 1.31 | -1.83 | -1.75 | 0.08 | 0.137 | 1.000 | 1.000 | 1.000 | 1.000 | 1.000 | 20 | 37 | 43 | 89 |
| **Vibrionaceae** | 1.95 | 0.92 | -0.79 | -1.04 | -2.74 | -1.70 | 0.537 | 1.000 | 1.000 | 1.000 | 0.539 | 1.000 | 28 | 33 | 131 | 70 |
| **DSM-21967** | 3.56 | -0.68 | -0.17 | -4.24 | -3.73 | 0.51 | 0.177 | 1.000 | 1.000 | 0.095 | 0.225 | 1.000 | 44 | 265 | 29 | 69 |

Table S11: Results from ANCOMBC analysis showing differentially abundant microbial families between treatments at TEnd. Log fold changes are on the natural log scale and indicate differences in antibiotic treated sponges when compared to control sponges. . * = p < 0.05, ** = p < 0.01.

|  | **Log_2_ fold changes** | | | | | | **BH corrected p-value** | | | | | | **Total Read Counts** | | | |
| --- | --- | --- | --- | --- | --- | --- | --- | --- | --- | --- | --- | --- | --- | --- | --- | --- |
| **Family** | **CTRL-**  **HW** | **CTRL-**  **AB** | **CTRL-**  **AH** | **HW-**  **AB** | **HW-**  **AH** | **AB-**  **AH** | **CTRL-**  **HW** | **CTRL-**  **AB** | **CTRL-**  **AH** | **HW-**  **AB** | **HW-**  **AH** | **AB-**  **AH** | **CTRL** | **HW** | **AB** | **AH** |
| **Rhodospirillaceae** | -2.50 | -13.08 | -12.95 | -10.58 | -10.45 | 0.12 | 1.000 | **0.001**** | **0.001**** | **0.002**** | **0.004**** | 1.000 | 74013 | 143178 | 26 | 81 |
| **NAC60-12** | -3.29 | -15.10 | -13.19 | -11.80 | -9.89 | 1.90 | 1.000 | **0.001**** | **0.001**** | **0.000**** | **0.005**** | 1.000 | 84349 | 137879 | 10 | 344 |
| **Thiohalobacteraceae** | -2.40 | -7.14 | -8.74 | -4.735 | -6.33 | -1.59 | 1.000 | **0.001**** | **0.001**** | 0.123 | **0.035*** | 1.000 | 5952 | 4159 | 156 | 198 |
| **Tenderiaceae** | -2.96 | -4.04 | -5.29 | -1.081 | -2.32 | -1.24 | 0.286 | **0.004**** | **0.003**** | 1.000 | 1.000 | 1.000 | 255 | 150 | 72 | 82 |
| **Sphingomonadaceae** | -3.35 | -5.13 | -3.89 | -1.78 | -0.54 | 1.23 | 1.000 | **0.017*** | 0.925 | 1.000 | 1.000 | 1.000 | 1459 | 590 | 74 | 1719 |
| **Endozoicomonadaceae** | -1.94 | -4.33 | -4.27 | -2.38 | -2.32 | 0.05 | 1.000 | **0.024*** | **0.037*** | 1.000 | 1.000 | 1.000 | 339 | 1556 | 110 | 191 |
| **GLO-3** | -2.99 | -6.13 | -10.74 | -3.14 | -7.75 | -4.60 | 1.000 | **0.030*** | **0.001**** | 1.000 | **0.002**** | 0.128 | 9517 | 11333 | 1101 | 14 |
| **Nitrosopumilaceae** | -3.61 | -1.96 | -2.97 | 1.64 | 0.63 | -1.01 | 1.000 | 0.838 | 0.081 | 1.000 | 1.000 | 1.000 | 499766 | 392762 | 422566 | 910380 |
| **Akkermansiaceae** | -2.60 | -1.89 | -6.19 | 0.70 | -3.59 | -4.30 | 0.472 | 1.000 | **0.001**** | 1.000 | 0.454 | 0.233 | 979 | 1005 | 1970 | 258 |
| **Francisellaceae** | 3.43 | 1.07 | 2.062 | -2.35 | -1.36 | 0.98 | 0.117 | 1.000 | 1.000 | 1.000 | 1.000 | 1.000 | 6 | 326 | 66 | 585 |
| **Ga0077536** | -1.38 | -0.58 | -2.07 | 0.77 | -0.71 | -1.49 | 1.000 | 1.000 | 0.413 | 1.000 | 1.000 | 1.000 | 101640 | 127638 | 178649 | 231094 |

Table S12: Table of results from post-hoc pairwise analyses of differences in mean respiration rate between sponges under different treatments at each time point. * = p < 0.05, ** = p < 0.01.

| Time | Contrast | Estimate | SE | DF | T Ratio | P (BH) |
| --- | --- | --- | --- | --- | --- | --- |
| T1 | Antibiotic vs. Control | 0.0053 | 0.0343 | 28.2523 | 0.1536 | 0.9084 |
| T2 | Antibiotic vs. (Antibiotic + Heatwave) | -0.1089 | 0.0492 | 91.6794 | -2.2129 | 0.0899 |
|  | Antibiotic vs. Control | 0.0074 | 0.0502 | 52.8454 | 0.1480 | 0.9084 |
|  | Antibiotic vs. Heatwave | -0.0747 | 0.0502 | 52.8454 | -1.4880 | 0.2973 |
|  | (Antibiotic + Heatwave) vs. Control | 0.1164 | 0.0502 | 52.8454 | 2.3166 | 0.0873 |
|  | (Antibiotic + Heatwave) vs. Heatwave | 0.0342 | 0.0502 | 52.8454 | 0.6806 | 0.7319 |
|  | Control vs. Heatwave | -0.0822 | 0.0492 | 91.6794 | -1.6694 | 0.2238 |
| T3 | Antibiotic vs. (Antibiotic + Heatwave) | -0.0268 | 0.0492 | 91.6794 | -0.5451 | 0.7338 |
|  | Antibiotic vs. Control | -0.0168 | 0.0502 | 52.8454 | -0.3349 | 0.8798 |
|  | Antibiotic vs. Heatwave | 0.0277 | 0.0502 | 52.8454 | 0.5523 | 0.7338 |
|  | (Antibiotic + Heatwave) vs. Control | 0.0100 | 0.0502 | 52.8454 | 0.1993 | 0.9084 |
|  | (Antibiotic + Heatwave) vs. Heatwave | 0.0546 | 0.0502 | 52.8454 | 1.0864 | 0.5427 |
|  | Control vs. Heatwave | 0.0446 | 0.0492 | 91.6794 | 0.9052 | 0.6391 |
| T4 | Antibiotic vs. (Antibiotic + Heatwave) | -0.2113 | 0.0492 | 91.6794 | -4.2927 | 0.0005** |
|  | Antibiotic vs. Control | 0.0441 | 0.0502 | 52.8454 | 0.8788 | 0.6391 |
|  | Antibiotic vs. Heatwave | -0.1133 | 0.0517 | 57.4475 | -2.1930 | 0.0899 |
|  | (Antibiotic + Heatwave) vs. Control | 0.2554 | 0.0502 | 52.8454 | 5.0856 | 0.0001** |
|  | (Antibiotic + Heatwave) vs. Heatwave | 0.0980 | 0.0517 | 57.4475 | 1.8974 | 0.1570 |
|  | Control vs. Heatwave | -0.1574 | 0.0507 | 96.5093 | -3.1063 | 0.0207* |
| TEnd | Antibiotic vs. (Antibiotic + Heatwave) | -0.0313 | 0.0492 | 91.6794 | -0.6351 | 0.7319 |
|  | Antibiotic vs. Control | -0.0371 | 0.0502 | 52.8454 | -0.7380 | 0.7246 |
|  | Antibiotic vs. Heatwave | -0.1590 | 0.0535 | 62.4602 | -2.9741 | 0.0261* |
|  | (Antibiotic + Heatwave) vs. Control | -0.0058 | 0.0502 | 52.8454 | -0.1106 | 0.9084 |
|  | (Antibiotic + Heatwave) vs. Heatwave | -0.1277 | 0.0535 | 62.4602 | -2.3893 | 0.0873 |
|  | Control vs. Heatwave | -0.1219 | 0.0525 | 100.6325 | -2.3216 | 0.0873 |

Table S13: Results from multivariate GLM showing correlations between individual ASV read counts and respiration rate. ASVs listed are those which were significantly correlated with respiration rate before p-value correction. . * = p < 0.05, ** = p < 0.01.

| ASV | Slope | SE | P-Value (BH) | Family |
| --- | --- | --- | --- | --- |
| Zotu100 | -8.32675 | 2.299596 | 0.223043 | *SOKP01* |
| Zotu6 | 3.53248 | 1.860021 | 0.223043 | *NAC60-12* |
| Zotu20 | -3.44035 | 1.411713 | 0.223043 | *Akkermansiaceae* |
| Zotu48 | 5.815029 | 1.697711 | 0.223043 | *Schleiferiaceae* |
| Zotu65 | 5.53805 | 2.097161 | 0.223043 | *Alteromonadaceae* |
| Zotu923 | 2.895549 | 1.907012 | 0.223043 | *GLO-3* |
| Zotu79 | -3.23028 | 1.336939 | 0.223043 | *Cyanobiaceae* |
| Zotu1250 | 2.185456 | 1.09345 | 0.223043 | *Tenderiaceae* |
| Zotu1365 | 3.301719 | 1.614823 | 0.223043 | *Ga0077536* |
| Zotu979 | 2.626349 | 1.928016 | 0.223043 | *Rhodospirillaceae* |
| Zotu94 | 5.557936 | 2.882856 | 0.223043 | *Desulfobacteraceae* |
| Zotu46 | -3.54749 | 1.447937 | 0.223043 | *UBA8477* |
| Zotu58 | 7.134867 | 2.700213 | 0.223043 | *UBA2595* |
| Zotu15 | 8.104352 | 1.469353 | 0.223043 | *Schleiferiaceae* |
| Zotu30 | 4.916 | 1.361932 | 0.223043 | *Schleiferiaceae* |
| Zotu43 | 7.386746 | 2.332855 | 0.223043 | *Flavobacteriaceae* |
| Zotu144 | 2.997847 | 1.804955 | 0.223043 | *Rhodospirillaceae* |
| Zotu18 | 7.64735 | 2.00357 | 0.223043 | *Alteromonadaceae* |

Table S14: Results from multivariate GLM showing correlations between individual family read counts and respiration rate. Families listed are those which were significantly correlated with respiration rate before p-value correction. . * = p < 0.05, ** = p < 0.01.

| Family | Slope | SE | P-Value (BH) |
| --- | --- | --- | --- |
| SOKP01 | -3.29069 | 1.373713 | 0.046857* |
| Akkermansiaceae | -2.92001 | 1.099134 | 0.046857* |
| Rhodospirillaceae | 2.163687 | 1.573201 | 0.046857* |
| UBA8477 | -3.54749 | 1.447937 | 0.046857* |
| Desulfobacteraceae | 5.557936 | 2.882856 | 0.046857* |
| UBA2595 | 5.279001 | 2.061959 | 0.046857* |
| Alteromonadaceae | 5.96529 | 1.33114 | 0.046857* |
| Schleiferiaceae | 7.033484 | 1.406884 | 0.049* |
